# A Reinforcement Learning-Guided Genetic Algorithm Integrating Medicinal Chemistry-Inspired Molecular Transformations

**DOI:** 10.1021/acs.jcim.6c00397

**Published:** 2026-04-16

**Authors:** Domenico Alberga, Vittoria Nanna, Maria Giovanna Eva Papadopoulos, Adolfo Ancona, Maria Cristina Lomuscio, Giuseppe Felice Mangiatordi

**Affiliations:** † 9327 CNR − Institute of Crystallography, Via Amendola 122/o, Bari 70126, Italy; ‡ Department of Biotechnology, Chemistry and Pharmacy, University of Siena, Siena 53100, Italy; § Dipartimento di Medicina di Precisione e Rigenerativa e Area Jonica (DiMePRe-J), Università Degli Studi di Bari Aldo Moro, Piazza Giulio Cesare, 11, Policlinico, Bari 70124, Italy

## Abstract

Achieving optimal target activity while maintaining synthetic accessibility and drug-likeness represents a major challenge in computational drug discovery. Existing *de novo* generative models often yield chemically invalid or synthetically intractable structures and struggle to optimize multiple objectives simultaneously. Here, we introduce ALCHIMIA, an interpretable hybrid framework combining reinforcement learning (RL) and a genetic algorithm (GA), built based on a vocabulary of 33 medicinal chemistry-inspired molecular transformations. The RL component trains a policy network to prioritize transformation sequences that improve synthetic accessibility (SA) and the quantitative estimate of drug-likeness (QED) scores, embedding these constraints directly into molecular generation. The GA component applies the learned policy as a mutational operator within population-based optimization guided by molecular docking, enabling the exploration of diverse chemical lineages while converging toward high-affinity ligands. ALCHIMIA was applied to two different pharmacologically relevant targets: human Cannabinoid Receptor 2 (CB2R) and human Sigma nonopioid intracellular Receptor 1 (S1R). We considered three different scenarios: (i) unconstrained hit identification; (ii) scaffold-constrained lead optimization; and (iii) design of dual modulators. The framework generated chemically valid molecules with QED and SA scores comparable to or better than those obtained with random baselines and selected *de novo* design methods. By codifying typical medicinal chemistry actions as learnable transformations and coupling multiobjective optimization with GA-based diversity maintenance, ALCHIMIA, freely available as a GitHub repository (https://github.com/alberdom88/ALCHIMIA), provides a practical, interpretable, and scalable framework for molecular *de novo* design.

## Graphical abstract

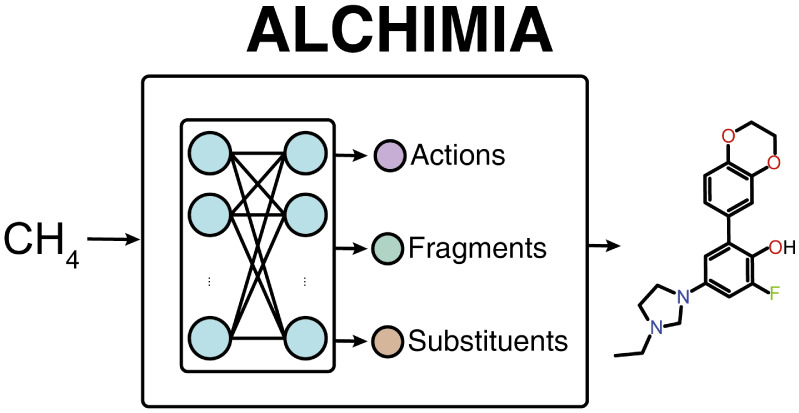

## Introduction

Drug discovery remains one of the most challenging and resource-intensive scientific endeavors,^1^ reflecting the intrinsic difficulty of identifying chemicals that concurrently satisfy stringent requirements, including high target affinity and selectivity, favorable absorption-distribution-metabolism-excretion-toxicity (ADMET) profiles, synthetic accessibility (SA), and suitable pharmacokinetic properties for the intended route of administration.^2^ Traditional medicinal chemistry relies on iterative cycles of design, synthesis, and biological testing, typically evaluating only hundreds to thousands of compounds per project—a tiny fraction of the estimated 10^23^ to 10^60^ drug-like molecules in the chemical space.^3^ This disparity has driven the development of computational approaches to accelerate hit identification and lead optimization phases, the stages most amenable to algorithmic intervention.^4–6^ The advent of artificial intelligence (AI), and more specifically deep learning (DL), has catalyzed a paradigm shift in computer-aided drug design (CADD), promoting the transition from the development and application of predictive models (e.g., Quantitative Structure-Activity Relationships - QSAR) toward data-driven generative models (*de novo* design) capable of proposing novel chemical scaffolds.^7–11^

*De novo* molecular design—the algorithmic generation of novel chemical structures optimized for desired property profiles—has emerged as a central research focus, with generative models learning to navigate chemical space by capturing statistical patterns from large molecular databases.^7^ Early neural architectures, including recurrent neural networks (RNNs), variational autoencoders (VAEs), and generative adversarial networks (GANs), demonstrated proof-of-concept for learning molecular representations and sampling novel structures, though they often struggled with issues including training instability, mode collapse, and the generation of chemically invalid structures.^12^ Transformer architectures, originally developed for natural language processing, have more recently achieved state-of-the-art performance in molecular generation by capturing long-range dependencies in SMILES strings or molecular graphs, with generative pretrained transformer (GPT)-based models and junction tree VAEs demonstrating improved validity and diversity over earlier approaches.^9,11^ However, such models often replicate training set distributions without any optimization.^12,13^ Reinforcement learning (RL) frameworks address this limitation and have demonstrated a great ability to guide generative policies toward desired property profiles via reward-based training.^14–17^ RL approaches face other challenges, such as (i) the low diversity of generated molecules, which arises from the small number of compounds predicted as active by the employed model (sparse rewards); (ii) training instability from high-variance policy gradients; and (iii) difficulty balancing exploration (discovering novel chemical scaffolds) versus exploitation (optimizing within known chemical neighborhoods).^13^ To overcome these limitations, new techniques have been developed, including experience replay, transfer learning from pretrained models, curriculum learning progressively increasing task difficulty, and multiagent architectures that enable parallel exploration of diverse chemical strategies.^14,15^

Despite the proliferation of deep generative models, several critical challenges remain inadequately addressed by current state-of-the-art methods. First, synthetic accessibility is often treated as a *post hoc* filter or weak penalty term rather than being fundamentally integrated into the generation process, resulting in molecules that achieve impressive *in silico* property predictions but prove synthetically intractable upon experimental pursuit.^16^ Second, dual-target and polypharmacology design—increasingly recognized as essential for treating complex multifactorial diseases—remains underexplored, with most methods optimizing single-target affinity despite growing evidence that balanced multitarget modulation can achieve superior efficacy and reduced resistance.^17–19^ Third, scaffold control enabling both unconstrained hit discovery and similarity-constrained lead optimization within a unified framework is rarely achieved, forcing practitioners to employ different tools for different optimization scenarios. Fourth, interpretability and medicinal chemistry intuition are often sacrificed in favor of black-box neural architectures, limiting adoption by medicinal chemists who require transparent rationales for proposed modifications.^4^

An alternative paradigm for molecular optimization combines evolutionary algorithms with structure-based fitness evaluation, leveraging population-based search to maintain chemical diversity while systematically improving specific objectives.^20^ In this context, evolutionary methods, such as those based on genetic algorithms (GAs), offer several conceptual advantages over purely generative approaches: population diversity mechanisms naturally balance exploration and exploitation, fitness-based selection provides clear optimization trajectories, and convergence detection criteria enable automatic termination once optimization potential is exhausted.^21^ Interestingly, recent GA-based approaches have introduced chemistry-aware mutation operators inspired by common medicinal chemistry transformations (e.g., bioisosteric replacement, functional group interconversion, scaffold hopping), which preserve synthetic feasibility.^22–26^ In this context, integrating RL with evolutionary algorithms represents an emerging frontier that combines the strengths of both paradigms: RL extracts generalizable transformation strategies from data, while GAs explore diverse molecular lineages guided by a predictive model such as molecular docking.^22^

Building on these foundations, herein, we present a hybrid computational framework named ALCHIMIA (A Reinforcement Learning-guided genetic algorithm Integrating Medicinal chemistry-Inspired moleculAr transformations) that addresses major limitations of current generative models by synergistically integrating RL with GA. ALCHIMIA employs a set of 33 medicinal chemistry-inspired molecular transformation operations as the fundamental vocabulary for molecular evolution. Our approach consists of two integrated components: (i) policy network training via RL that learns to prioritize transformation sequences optimizing SA^16^ and quantitative estimate of drug-likeness (QED)^27^ scores, and (ii) GA optimization that applies the learned policy to guide population-based exploration toward high-affinity binders via structure-based docking, with multicriteria convergence detection ensuring robust termination. The transformation operations, including aromatic decoration, functional group interconversion, bioisosteric replacement, scaffold hopping, and fragment addition/deletion, directly encode medicinal chemistry expertise, ensuring that all generated molecules lie within synthetically accessible chemical space by construction rather than by *post hoc* filtering. ALCHIMIA was tested on two targets involved in cancer and neurodegeneration, namely cannabinoid receptor 2 (CB2R)^28^ and sigma nonopioid intracellular receptor 1 (S1R),^29^ by taking into account three scenarios typically occurring in drug discovery projects: (i) single-target hit identification; (ii) dual-target hit identification; and (iii) lead optimization.

The results show that by codifying medicinal chemistry intuition into learnable transformation operations, while leveraging RL capacity for multiobjective optimization and GA population-based diversity maintenance, ALCHIMIA represents a practical and interpretable approach for *de novo* design.

## Methods

### Chemical Actions Definition

Our molecular generation framework employs a comprehensive set of 33 distinct mutation operations designed to explore drug-like chemical space through systematic molecular graph transformations. These operations can be categorized into six major classes: (i) fragment-based modifications; (ii) functional group transformations; (iii) ring system modifications; (iv) chemical reactions; (v) bioisosteric replacements; and (vi) chain modifications.

#### Fragment-Based Modifications

Operations that manage the assembly, removal, or replacement of entire structural blocks or complex substructures:

*1- ADD_FRAG:* It attaches molecular fragments, selected from a curated library of Breaking of Retro-synthetically Interesting Chemical Substructures (BRICS)-derived building blocks,^30^ at sites with available hydrogens. The BRICS library was obtained by computing the fragments from our test set (see the section “Test Set Preparation” for methodological details), retaining only those with a maximum of 10 heavy atoms. We ranked fragments by their occurrence and selected the top 0.1%, yielding a total of 468 fragments.

*2- DELETE_RING_SIDECHAIN:* It removes nonring side chains of a specified maximum size (12 heavy atoms) by identifying cuttable single bonds.

*3- REPLACE_SIDECHAIN_CORE:* It combines deletion and fragment attachment to enable scaffold hopping.

*4- REDUCE_CHIRALITY*: This action eliminates one stereocenter through hierarchical structural modifications based on three primary strategies: (A) removal of small side chains (≤12 heavy atoms; ≤3 atoms if ring-embedded) from chiral centers to identify fragment boundaries; (B) local unsaturation of sp^3^ carbons through double bond formation (sp^3^→sp^2^ conversions in C=O, C=N, and C=C patterns); and (C) pruning of exocyclic branches from quaternary chiral centers lacking a hydrogen atom. When direct reduction fails, a fallback strategy leads to a ring contraction or ring aromatization. The operation validates efficacy by ensuring that the final number of stereocenters is strictly lower than the original count, accepting modifications only when this criterion is met.

#### Functional Group Transformation

Modifications that alter the electronic/steric properties through the introduction or exchange of specific functional groups without altering the main connectivity of the scaffold:

*5- DECORATE_AROM:* It specifically targets aromatic carbons for decoration with small substituents (−F, −Cl, −Br, −I, −CF_3_, −NO_2_ and −SO_2_CH_3_).

*6- DELETE_DECOR_AROM*: It deletes the decoration, restoring the hydrogen bound to the aromatic carbon.

*7- REPLACE_DECOR_AROM:* It replaces an aromatic decoration with a different one.

*8- HALOGENATE*: This action introduces halogens (−F, −Cl, −Br, −I) at C–H sites, with a preference for aromatic positions.

*9- DEHALOGENATE*: It removes terminal halogens and replaces them with a hydrogen atom.

*10- SWAP_HALOGEN:* It exchanges one halogen for another at terminal positions.

*11- NITRATE*: It adds nitro groups (−NO_2_) onto aliphatic and aromatic rings.

*12- SULFONYLATE*: It introduces sulfonyl groups (−SO_2_R) preferentially at nucleophilic nitrogen sites to form sulfonamides or at aromatic carbons to form sulfones.

*13- ADD_POLAR_GROUP* and *ADD_LIPOPHILIC_GROUP*: These operations attach polar (−OH, −NH_2_, −COOH, −CONH_2_, −SO_2_NH_2_) or lipophilic (alkyl, aryl) substituents at sites with available hydrogens.

*14- SWAP_POLAR_LIPOPHILIC*: It exchanges one polar group (−OH, −NH_2_, −COOH, −CONH_2_, −SO_2_NH_2_) for one lipophilic group (alkyl, aryl) and vice versa.

*15- ALKYLATE_HETERO*: It specifically methylates or ethylates nucleophilic heteroatoms (N, O).

*16- METHYLATE_AMINE*: This action adds methyl groups to primary, secondary, or tertiary amines; for tertiary amines, methylation produces quaternary ammonium ions ((R)_3_-N^+^ −CH_3_).

*17- DEMETHYLATE_AMINE*: It removes terminal N–CH_3_ groups.

#### Ring System Modifications

Topological operations that create cycles alter their nature, size, or merging:

*18- SATURATE_RING*: It converts aromatic rings into saturated cycloalkanes by removing aromaticity flags and setting all ring bonds to single bonds.

*19- AROMATIZE_RING*: It attempts the reverse transformation on 6-membered carbocyclic rings by introducing alternating double bonds.

*20- EXPAND_RING*: It inserts a methylene group (−CH_2_−) into the ring bonds to create larger rings.

*21- CONTRACT_RING*: It removes a methylene group (−CH_2_−) and connects its neighbors to produce smaller rings.

*22- FUSE_RINGS:* It creates fused bicyclic systems by identifying ring edges with adjacent noncyclic chains (2–4 atoms) and forming new bonds between chain termini and the opposite ring atom, generating 5–7-membered fused rings.

*23- CYCLIZE_CHAIN*: It closes acyclic chains by connecting terminal heteroatoms (N, O) or carbons through single bonds when separated by 3–10 atoms along single-bond paths, avoiding ring formation across existing cycles.

#### Chemical Reactions

Operations that mimic specific synthetic reactions involving changes in oxidation state or the formation/hydrolysis of amide and ester bonds:

*24- OXIDIZE_ALCOHOL*: This action converts primary and secondary alcohols to carbonyl groups (R-CH_2_–OH → R-CHO, R–CH­(OH)-R’ → R–CO–R’) by transforming C–O single bonds into double bonds.

*25- REDUCE_CARBONYL:* It performs the reverse transformation on aldehydes and ketones, excluding esters and amides through neighbor analysis.

*26- HYDROLYZE_ESTER:* It cleaves ester bonds (R–CO–O–R’ → R-COOH + R’–OH) by breaking the acyl-oxygen bond and adding a hydroxyl group.

*27- FORM_ESTER* and *FORM_AMIDE*: These operations simulate condensation reactions by removing carboxyl groups and attaching external alcohol or amine fragments, respectively.

*28- ACETYLATE_PHENOL*: It acetylates phenols (Ar–OH → Ar–O–CO–CH_3_)

*29- DEACETYLATE*: It removes simple acetyl groups (R-CO–CH_3_) by identifying and deleting the complete acyl branch.

#### Bioisosteric Replacements

Targeted substitutions maintain biological activity by modifying the physicochemical properties.

*30- BIOISOSTERIC_SWAP*: It encompasses multiple substitution strategies, including ring heteroatom exchanges (-O- ↔ -NH- in 5-membered rings), linker atom swaps (−CH_2_– ↔ −NH– ↔ −O– ↔ −S−), carbonyl/thiocarbonyl interconversion (C=O ↔ C=S), ester/hydrazide exchange (R–CO–O–R’ ↔ R–CO–NH–NH–R’), terminal group substitutions (-F- ↔ -H, −OH ↔ −NH_2_ ↔ −CH_3_), and heavy halogen swaps (Cl ↔ Br ↔ I).

#### Chain Modifications

Modifications to the length and flexibility of aliphatic linkers:

*31- INSERT_CH2_LINKER*: It inserts methylene spacers into noncyclic single bonds between heavy atoms.

*32- DELETE_CH2_LINKER*: It removes sp^3^–CH_2_– groups that connect two heavy atoms.

In addition to these modifications, a stop operation (33-*STOP*) was incorporated into the algorithm to terminate the modification process upon selection. To improve robustness, operations are retried up to 20 times if the resulting structure does not pass the RDKit validity check.

### Reinforcement Learning Algorithm

ALCHIMIA employs an RL approach based on the REINFORCE algorithm,^31^ which is a Monte Carlo policy gradient method that directly optimizes a parameterized stochastic policy to maximize expected discounted cumulative rewards.^32^ The policy network architecture, schematized in Figure 1, consists of a feedforward neural network that processes molecular fingerprint representations (Morgan circular fingerprints with radius 2 and 2048 bits)^33^ and outputs discrete action distributions. Specifically, the network consists of a shared two-layer backbone (hidden dimension = 512 and Rectified Linear Unit (ReLU) activations) that extracts latent features from molecular fingerprints, followed by three specialized parallel heads: (i) an action head consisting of a three-layer network (hidden dimension = 512) producing logits over the 33 available molecular operations; (ii) a core fragment head consisting of a three-layer network (hidden dimension = 256) for selecting BRICS-derived scaffold fragments for operations requiring core attachments (ADD_FRAG, REPLACE_SIDECHAIN_CORE); and (iii) a decoration fragment head consisting of a three-layer network (hidden dimension = 256) for sampling small substituents for aromatic decoration operations (DECORATE_AROM, REPLACE_DECOR_AROM).

**Figure 1:**
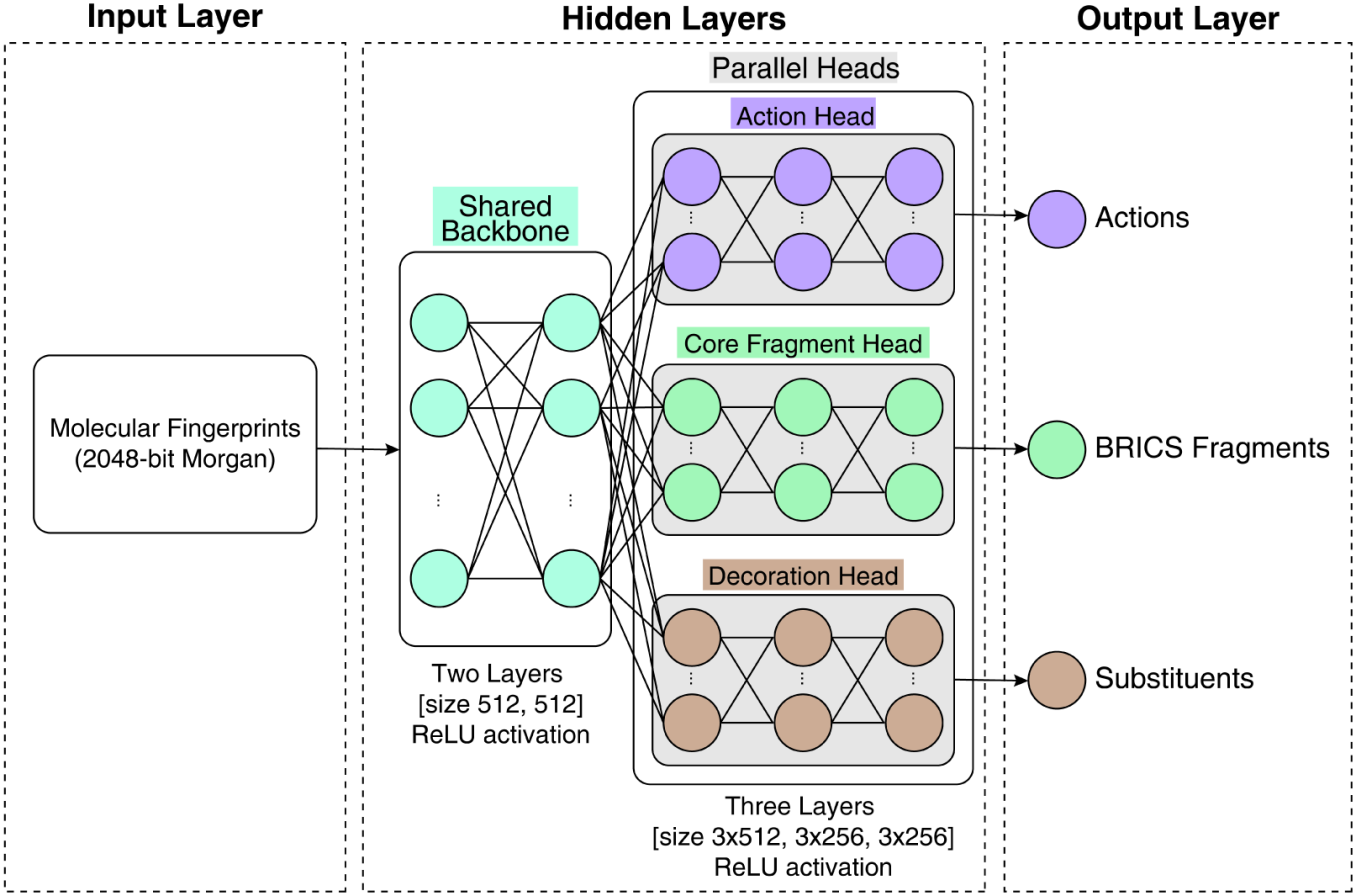
Workflow of the ALCHIMIA algorithm. The model presents a shared two-layer backbone (hidden dimension H = 512) feeding three parallel heads: the Action head (three layers, H = 512) for the selection of molecular operations, the Core Fragment head (three layers, H = 256) for scaffold selection, and a Decoration head (three layers, H = 256) for substituent sampling.

The training procedure follows an episodic paradigm, where each episode begins with a source molecule and iteratively applies sampled actions until either a STOP action is selected (prohibited at the first step to prevent trivial solutions) or a maximum step limit, namely M, is reached. At each time step *t*, the current molecular state *m_t_* is converted to its fingerprint representation *X*_t_, which is passed through the policy network to obtain action logits. Actions are sampled using temperature scaling (default 1.0)^34^ and top-p (nucleus) sampling (default 0.95),^35^ which softens the logit distribution and truncates the cumulative probability mass at threshold *p*, enabling controlled stochastic exploration while preventing sampling from the long tail of unlikely actions. The reward function *R* is designed to balance SA^16^ and QED^27^ scores:

$$R=0.5\cdot \overset{―}{SA}\left(m_{\mathrm{gen}}\right)+0.5\cdot \mathrm{QED}\left(m_{\mathrm{gen}}\right)$$

where *m*_gen_ is the generated molecule and

$\overset{―}{SA}=1.0-\left(SA-1.0\right)/9.0$ is a normalized synthetic accessibility score that maps the native SA range—from 1 (most synthetically accessible) to 10 (least accessible)—onto [0,1], such that higher values correspond to greater synthetic accessibility, consistent with the maximization objective of the reward function. Notice that a penalty of −0.05 is applied if the generated molecule is identical to the source to discourage no-operation trajectories. Policy optimization follows the REINFORCE gradient estimator with baseline subtraction^36^ (decay *α* = 0.9) to reduce variance in gradient estimates and entropy regularization^31^ (entropy coefficient *β* = 0.01) penalizing low-entropy (overly deterministic) policies to maintain exploration throughout training.^37^ The baseline is shared across all episodes from a source molecule and updated only after processing all episodes for that source. For each source molecule, 15 episodes are executed to accumulate gradients, which are averaged and applied via Adam optimization^38^ (learning rate 10^–4^) with gradient clipping at 1.0 to stabilize training.

Training employs a curriculum learning strategy^39,40^ initialized from methane (CH_4_) as the seed molecule (epoch 0). After each epoch, all unique generated molecules are appended to a cumulative training set. At each epoch *e*, the training set consists exclusively of 5,000 randomly selected molecules generated in the previous epoch (*e* – 1), implementing a progressive complexity expansion where the molecular space is explored incrementally from the initial seed. This iterative expansion process enables the model to progressively explore chemical space, starting from the simplest molecule and building complexity through learned transformations. Training proceeds for 100 epochs; model checkpoints are saved after each epoch, and a detailed CSV log records epoch-level statistics, including average total reward, Tanimoto similarity of the generated compounds with respect to the source molecules, SA scores, QED values, and average trajectory length.

Training was performed on a single NVIDIA H100 GPU, requiring approximately 11 h (M = 1) and 51 h (M = 9). Notably, once trained, the policy networks are lightweight and can be deployed on standard hardware: the generation of 1,000 molecules on a laptop equipped with an Intel i7–13700H processor and 32 GB RAM required only 8.6 s for M = 1 and 23.7 s for M = 9.

### Test Set Preparation

The test set was constructed from the ChEMBL database (version 35),^41^ a manually curated repository of bioactive molecules with experimentally measured drug-like properties maintained by the European Bioinformatics Institute. The initial data set comprised molecules with experimentally annotated binding affinities or functional activities, specifically those exhibiting K_i_ or IC_50_ values below 1 μM.^42^ A comprehensive molecular standardization pipeline available in the RDKit toolkit was used.^43^ The standardization workflow proceeded through the following sequential operations: (i) largest fragment selection using *rdMolStandardize.LargestFragmentChooser* with *preferOrganic = True* to isolate the primary organic component and remove counterions, solvents, and metal salts commonly present in crystallographic or formulation data; (ii) charge neutralization via *rdMolStandardize.Uncharger*, which removes formal charges by adjusting protonation states to generate neutral species, thereby eliminating pH-dependent ionization artifacts and ensuring uniform representation of ionizable functional groups; (iii) stereochemistry removal using *Chem.RemoveStereochemistry*; and (iv) isotope parent generation through *rdMolStandardize.IsotopeParent* to replace heavy isotopes (^2^H, ^13^C, etc.) with their natural abundance counterparts, eliminating isotopic labeling artifacts from tracer studies. After standardization, a filter based on the elemental composition was applied. In particular, only molecules composed exclusively of C, N, O, F, P, S, Cl, Br, and I were retained. A critical molecular weight filter was subsequently imposed, retaining only molecules with molecular weights in the range of 160–600 Da, thus excluding very small fragments and aligning with the extended Lipinski “Rule of Five” guidelines.^44^ After the application of all filtering and standardization criteria, the curated data set contained 373,448 unique molecules. The test set was finally created by randomly selecting 100,000 molecules.

### Genetic Algorithm

ALCHIMIA employs a GA that integrates the trained RL policy networks with a molecular docking-based evaluation. The GA operates on a population of candidate molecules that evolve over successive generations, exploiting the RL policy as a mutational operator and using fitness-based ranking to identify high-affinity ligands against a target protein. During each generation step, each molecule in the elite pool is processed by an RL model randomly chosen from three variants corresponding to M = 1, 2, or 3. The selected model applies up to ten M mutational operations to generate 10 new molecules. For each considered input molecule, the trained RL policy network generates proposed mutations by sampling an action from the policy distribution and executing the corresponding transformation.

Subsequently, all the generated molecules are subjected to docking simulations performed using Glide software^45^ and added to the global pool. Finally, the new elite pool selection proceeds through a fitness-based ranking mechanism with diversity preservation constraints to prevent premature convergence. Molecules are first ranked by ligand efficiency (LE), defined as the docking score (DS) divided by the square root of the number of heavy atoms.^46,47^ The top candidates are retained as elite members after passing a diversity filter based on Tanimoto similarity computed using the Morgan circular fingerprint with radius 2:^33^ starting from the highest-ranked molecule, a candidate is accepted only if its maximum similarity to any existing elite member is below a predefined threshold (sim_cut_), ensuring structural diversity. The elite pool size is capped at N = 20. If convergence is not reached, then this updated elite pool is used as input for the next generation cycle.

ALCHIMIA maintains two persistent molecular archives. The first stores the elite pool molecules from the current generation ranked by LE. The second accumulates all unique molecules evaluated throughout the entire optimization campaign, with their associated metrics (DS, LE, QED, SA, logP, and the number of chiral centers). Notably, the GA implemented in ALCHIMIA employs a multicriteria convergence detection framework that integrates four complementary termination conditions to balance computational efficiency with solution quality.

This stopping strategy operates by analyzing the statistical properties of the fitness score across successive generations and triggering termination when at least three of four predefined criteria are simultaneously satisfied.^48,49^ The first criterion detects sustained stagnation in the best (lowest) fitness score by tracking the number of consecutive generations without an improvement in the global minimum. This criterion is satisfied when no new best minimum is found for 25 consecutive generations. The second criterion quantifies the improvement in the population mean fitness across generations. This criterion is satisfied when the mean DS fails to improve by at least 0.1 kcal/mol for 25 consecutive generations. The third convergence criterion is defined in terms of the population size. The algorithm records the maximum population size attained across all generations. Convergence is considered achieved when the population decreases below the threshold of 100 individuals and remains under this limit for 25 consecutive generations. This requirement ensures that termination occurs only after sustained stabilization, thereby mitigating the risk of premature convergence. The fourth criterion ensures sufficient evolutionary time by permitting convergence only after 100 generations, thus avoiding the risk of premature convergence due to starting conditions.

### Molecular Docking

The generated compounds were docked against the X-ray structures of CB2R in complex with the agonist AM12033 (resolution: 3.20 Å; PDB ID: 6KPC)^50^ and S1R in complex with (+)-pentazocine (resolution: 3.12 Å; PDB ID: 6DK1),^51^ adapting the docking protocols from our previous works.^52–54^ The retrieved PDB files were processed using the Protein Preparation Workflow^55^ available in Schrödinger Suite 2025–2 to add missing hydrogens, rebuild incomplete side chains, assign optimal protonation states at physiological pH, remove crystallographic water molecules, optimize hydrogen-bonding networks, and perform force-field-based minimization with OPLS-4.^56^ Ligands were prepared using LigPrep^57^ available in Schrödinger Suite 2025–2, to remove salts and generate all relevant tautomers and ionization states at pH 7.0 ± 2.0. Docking simulations were carried out using Grid-based ligand docking with energetics (GLIDE)^58^ with the protein kept rigid and full ligand flexibility allowed. A cubic grid was centered on the cognate ligand, with an inner box of 10 Å × 10 Å × 10 Å for both proteins and outer boxes of 24.6 Å × 24.6 Å × 24.6 Å (6KPC) and 23.5 Å × 23.5 Å × 23.5 Å (6DK1). All simulations employed the OPLS_2005 force field^59^ and the standard precision (SP) docking protocol. Protocol validity was confirmed by redocking the cognate ligands, yielding RMSD values of 0.68 Å for AM12033 and 0.29 Å for (+)-pentazocine.

## Results

### Model Training

ALCHIMIA was trained using a curriculum learning paradigm initialized from CH_4_, progressively expanding the molecular space across 100 epochs and optimizing both SA and QED scores. The policy network’s performance was systematically evaluated across trajectory lengths ranging from M = 1 to M = 9 (where M is the number of sequential molecular transformation steps per episode) on both molecules generated in the last training epoch (training set) and a test set consisting of 100,000 molecules randomly selected from the filtered ChEMBL database. The quality metrics returned by molecules generated by ALCHIMIA from the training set across trajectory lengths from M = 1 to M = 9 are reported in Table 1.

**Table 1:** Quality Metrics for Molecules Generated by ALCHIMIA for Different Values of Trajectory Length M from (i) the Training Set, (ii) the Test Set, and (iii) the Test Set Obtained from the Test Set Using Random Actions^a^

M	Unicity (%)	sim	SA	QED
**Training Set**				
**1**	51.66	0.64 ± 0.13	3.19 ± 0.77	0.79 ± 0.12
**2**	73.01	0.62 ± 0.22	3.06 ± 0.76	0.83 ± 0.09
**3**	78.74	0.57 ± 0.24	3.07 ± 0.83	0.82 ± 0.10
**4**	81.69	0.52 ± 0.25	3.03 ± 0.83	0.83 ± 0.10
**5**	81.59	0.51 ± 0.25	3.01 ± 0.84	0.84 ± 0.10
**6**	82.28	0.48 ± 0.25	2.95 ± 0.88	0.83 ± 0.10
**7**	83.16	0.45 ± 0.25	2.91 ± 0.88	0.83 ± 0.11
**8**	80.98	0.43 ± 0.25	2.90 ± 0.92	0.82 ± 0.11
**9**	82.06	0.43 ± 0.25	2.83 ± 0.87	0.83 ± 0.11
**Test set**				
**1**	38.41	0.73 ± 0.12	2.92 ± 0.69	0.60 ± 0.19
**2**	67.28	0.61 ± 0.18	2.93 ± 0.72	0.64 ± 0.18
**3**	76.51	0.56 ± 0.20	2.95 ± 0.75	0.65 ± 0.17
**4**	80.59	0.48 ± 0.20	2.90 ± 0.77	0.68 ± 0.16
**5**	79.27	0.43 ± 0.19	2.85 ± 0.78	0.71 ± 0.15
**6**	81.47	0.40 ± 0.19	2.86 ± 0.81	0.71 ± 0.15
**7**	81.47	0.36 ± 0.19	2.81 ± 0.82	0.72 ± 0.15
**8**	79.32	0.35 ± 0.19	2.79 ± 0.83	0.71 ± 0.15
**9**	79.12	0.33 ± 0.18	2.78 ± 0.84	0.73 ± 0.14
**Random Actions**				
**1**	100.00	0.73 ± 0.13	3.20 ± 0.71	0.49 ± 0.20
**2**	100.00	0.66 ± 0.16	3.29 ± 0.74	0.48 ± 0.20
**3**	100.00	0.59 ± 0.18	3.38 ± 0.76	0.46 ± 0.20
**4**	100.00	0.53 ± 0.18	3.49 ± 0.78	0.44 ± 0.21
**5**	100.00	0.47 ± 0.17	3.59 ± 0.80	0.43 ± 0.21
**6**	100.00	0.43 ± 0.16	3.69 ± 0.82	0.41 ± 0.21
**7**	100.00	0.40 ± 0.15	3.78 ± 0.83	0.40 ± 0.21
**8**	100.00	0.37 ± 0.14	3.87 ± 0.84	0.38 ± 0.21
**9**	100.00	0.34 ± 0.14	3.96 ± 0.85	0.37 ± 0.21

a Unicity quantifies the fraction of structurally unique molecules; Tanimoto similarity (sim) represents the mean Tanimoto similarity (Morgan fingerprints, radius 2) between generated compounds and source molecules; SA denotes Synthetic Accessibility score;^16^ QED represents drug-likeness.^27^

Data reveal systematic patterns in policy network learning and chemical space metrics. Notably, the computed unicity, quantifying the fraction of structurally unique molecules, shows a characteristic plateau, increasing from ∼52% at M = 1 to peak values of 82–83% at M = 6–7, followed by a slight decline at M = 8 and M = 9. This trend reflects saturation effects, whereby additional sequential transformations within a bounded exploration space converge toward similar chemical modifications. Moreover, the rapid rise from M = 1 (51.66%) to M = 2 (73.01%) indicates that adding a second modification substantially increases structural variability, suggesting that the policy network rapidly learns to combine transformations. The averaged Tanimoto similarity (sim) between each generated compound and its corresponding predecessor shows a clear monotonic decline from 0.64 ± 0.13 at M = 1 to 0.43 ± 0.25 at M = 9. The relatively high standard deviations (0.22–0.25) throughout all trajectory lengths indicate substantial heterogeneity in individual trajectories, consistent with the stochastic action sampling that permits both conservative single-atom modifications and extensive multioperation transformations. Even at the minimal M = 1, the mean Tanimoto similarity of 0.64 indicates moderate structural divergence from source molecules, suggesting that even a single transformation productively accesses meaningful chemical neighborhoods rather than trivial single-atom perturbations.

The computed SA scores indicate a remarkable stability across all trajectory lengths, ranging narrowly from 3.19 ± 0.77 (M = 1) to 2.83 ± 0.87 (M = 9), indicating the ability of ALCHIMIA to generate molecules with good synthetic accessibility. Such stability is particularly meaningful given that Tanimoto similarity decreases by 0.21 units from M = 1 to M = 9, yet SA scores remain essentially constant. This suggests that the policy network has successfully learned to prioritize chemically reasonable transformations that preserve or modestly improve synthetic feasibility. The consistent SA values < 3.0 confirm systematic avoidance of synthetically challenging transformations (*e.g.;* strained rings and excessive stereo-complexity). As for the returned QED scores, consistent performance is observed across all trajectory lengths, with a mean value of 0.79 ± 0.12 at M = 1 remaining essentially stable regardless of the considered M, thus indicating that the learned policy successfully prioritizes transformation sequences that navigate toward drug-like, synthetically feasible chemical space. The tight standard deviations (0.09–0.11) indicate narrow QED distributions centered on drug-like values, suggesting that the explicit drug-likeness reward term drives the policy optimization into pharmacologically privileged regions of the chemical space.

Table 1 also reports the corresponding metrics obtained on the test set (100,000 randomly selected ChEMBL molecules), enabling an evaluation of the generalization of the learned policy when applied to real-life molecular scaffolds. Note that for each test set compound, 15 molecules were generated. The results mirror the trends observed for the training set: unicity increases from ∼38% at M = 1 to a plateau of 79–81% at M = 5–7, while the Tanimoto similarity decreases from 0.73 ± 0.12 to 0.33 ± 0.18. Critically, SA scores remain stable at 2.95–2.78 and QED at 0.60–0.73 across test trajectories. The consistent train-test alignment across all metrics indicates generalizable learning of transferable transformation strategies rather than memorization of training set-specific patterns, validating the policy network’s capacity for deployment on novel target molecules.

Furthermore, a critical ablation study was conducted comparing policy-guided generation with uniform random sampling from the 33 transformation operations, performed without learned policy guidance, in order to quantify the incremental value contributed by RL beyond stochastic exploration. This baseline is particularly instructive for elucidating how RL’s explicit optimization of molecular properties translates into practical advantages. While random transformations maintain moderate Tanimoto similarity (0.74–0.34 across trajectory lengths), random sampling leads to systematically degraded SA scores (3.20–3.87 vs 2.78–3.06 for policy-guided generation), reflecting the accumulation of synthetically unfavorable modifications, including strained ring systems, excessive stereochemical complexity, and challenging functional group arrangements that are difficult to address experimentally. Similarly, QED values decrease substantially (0.38–0.49 vs 0.64–0.83 for policy-guided generation), indicating the presence of molecules that violate Lipinski’s Rule of Five through excessive molecular weight, suboptimal logP, and polar surface area deviations. In contrast, the learned policy achieves higher molecular quality by prioritizing transformations that both preserve key chemical neighborhoods and improve pharmaceutical desirability—a balance essential for hit discovery (which requires novelty) and lead optimization (which requires scaffold retention).^60^

### Genetic Algorithm Applications

ALCHIMIA was applied to two pharmaceutically relevant targets implicated in cancer and neurodegeneration, namely CB2R and S1R, and assessed across two key drug-discovery scenarios: *de novo* hit identification and lead optimization. Notably, its capability to generate promising dual CB2R/S1R hits was also challenged, given the therapeutic potential of simultaneously modulating this target pair.^28^

#### Application to Hit Identification

ALCHIMIA was applied to multiple hit identification scenarios aimed at identifying new hit compounds targeting: *i)* CB2R; *ii)* S1R; and *iii)* both CB2R and S1R simultaneously. In all cases, molecular evolution started from CH_4_ as the sole seed molecule, enabling fully unconstrained exploration of chemical space and was driven solely by the learned RL policy as the mutational operator and by docking-based fitness evaluation under identical diversity and stopping criteria. In all cases, sim_cut_ was fixed at 0.4 to maximize structural diversity and enforce scaffold retention while exploring decoration patterns. This unified setup enables a direct comparison of how the same policy-guided GA explores the chemical space across distinct receptor topologies and a more stringent dual-target objective. These differences are reflected in the convergence profiles (Figure 2) and in the distribution, in the resulting libraries, of the physicochemical and drug-likeness metrics (Figures 3–5, Table 2). Across all three campaigns, the GA rapidly escaped the trivial methane-derived starting chemical space and converged, generating high-affinity, drug-like, and synthetically accessible ligands, with multicriteria termination consistently occurring after sustained plateauing of both best and mean fitness. Note that the three convergence profiles exhibited distinct qualitative behaviors that mirror the structural specificity of the respective targets.

**Figure 2:**
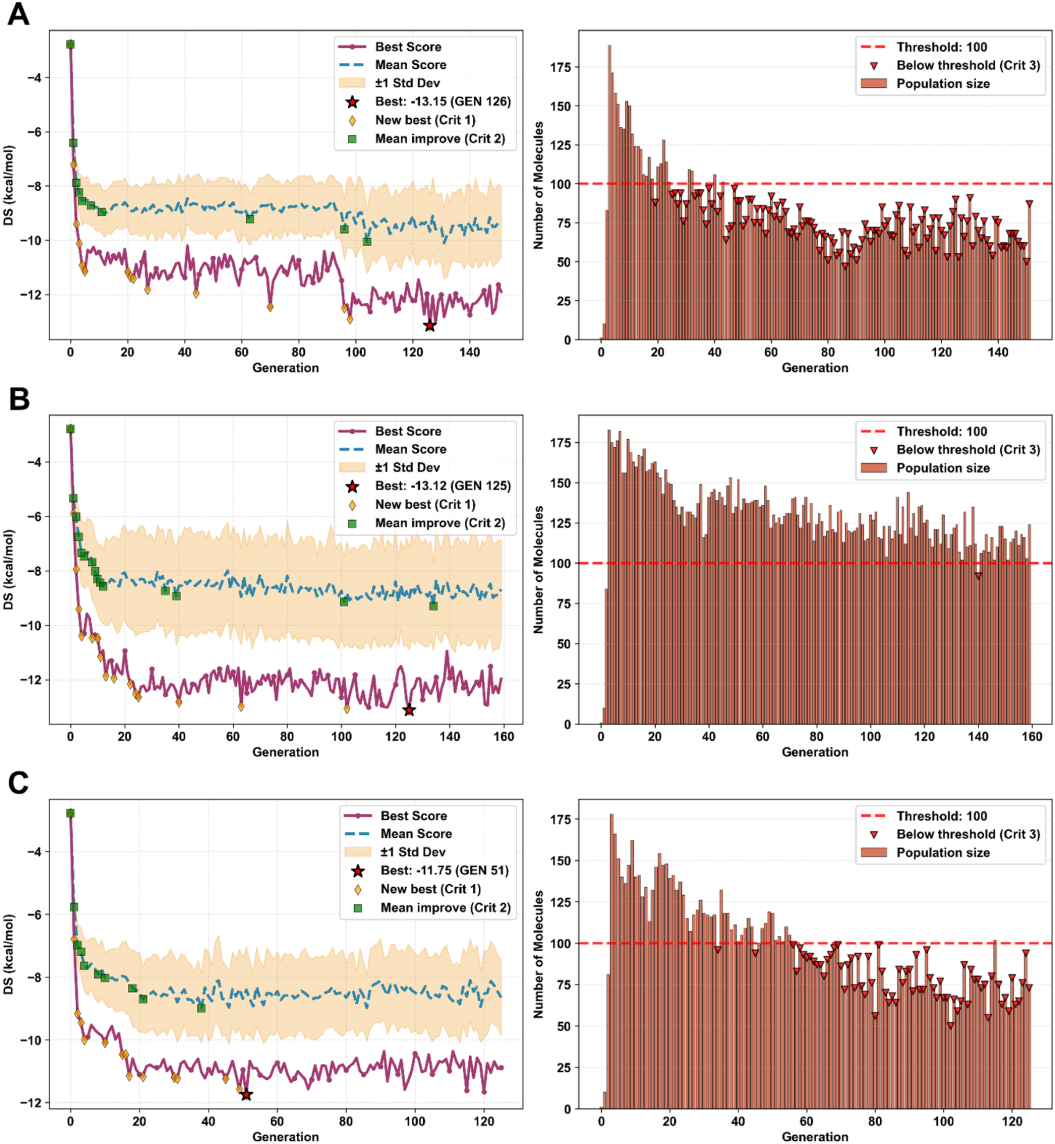
Genetic algorithm convergence profiles for hit identification across three distinct optimization scenarios: A) CB2R, B) S1R, and C) CB2R/S1R dual target. For each generation, the panels on the left show the best and mean DSs of the evolving population as purple and blue lines, respectively. Yellow diamonds indicate generations where a new global minimum is first observed, while green squares indicate generations where a new best mean score is obtained. The panels on the right report the population size per generation, with red triangles marking generations in which the population drops below the threshold of 100, highlighted by a horizontal red dashed line.

**Figure 3:**
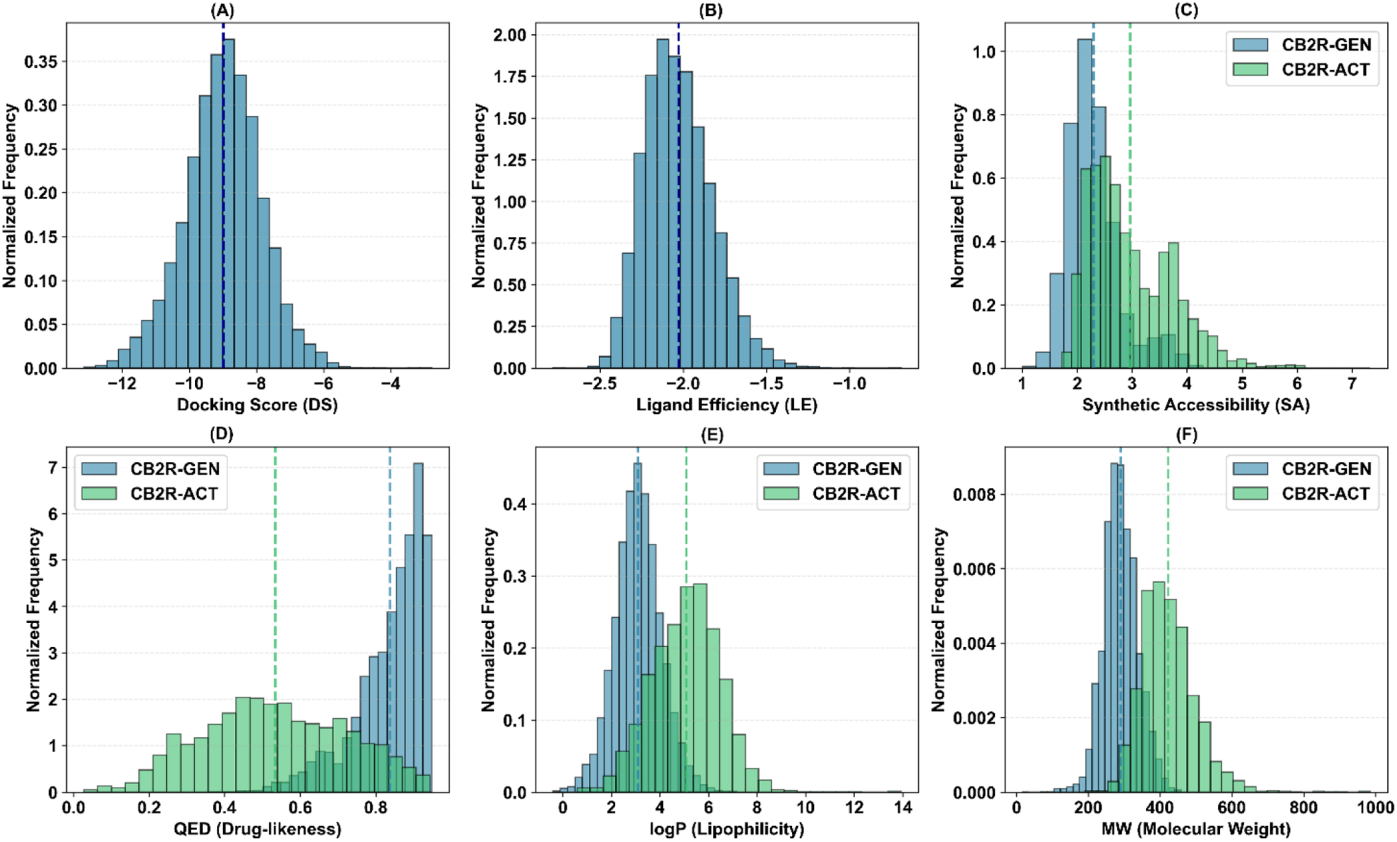
Property distributions for the CB2R hit identification campaign: A) docking score (DS), B) ligand efficiency (LE), C) synthetic accessibility (SA), D) quantitative estimate of drug-likeness (QED), E) lipophilicity (logP), and F) molecular weight (MW). Distributions for generated compounds (CB2R–GEN) and known actives found in ChEMBL v36 (CB2R–ACT) are colored in blue and green, respectively. Mean values are reported via vertical dashed lines.

**Figure 4:**
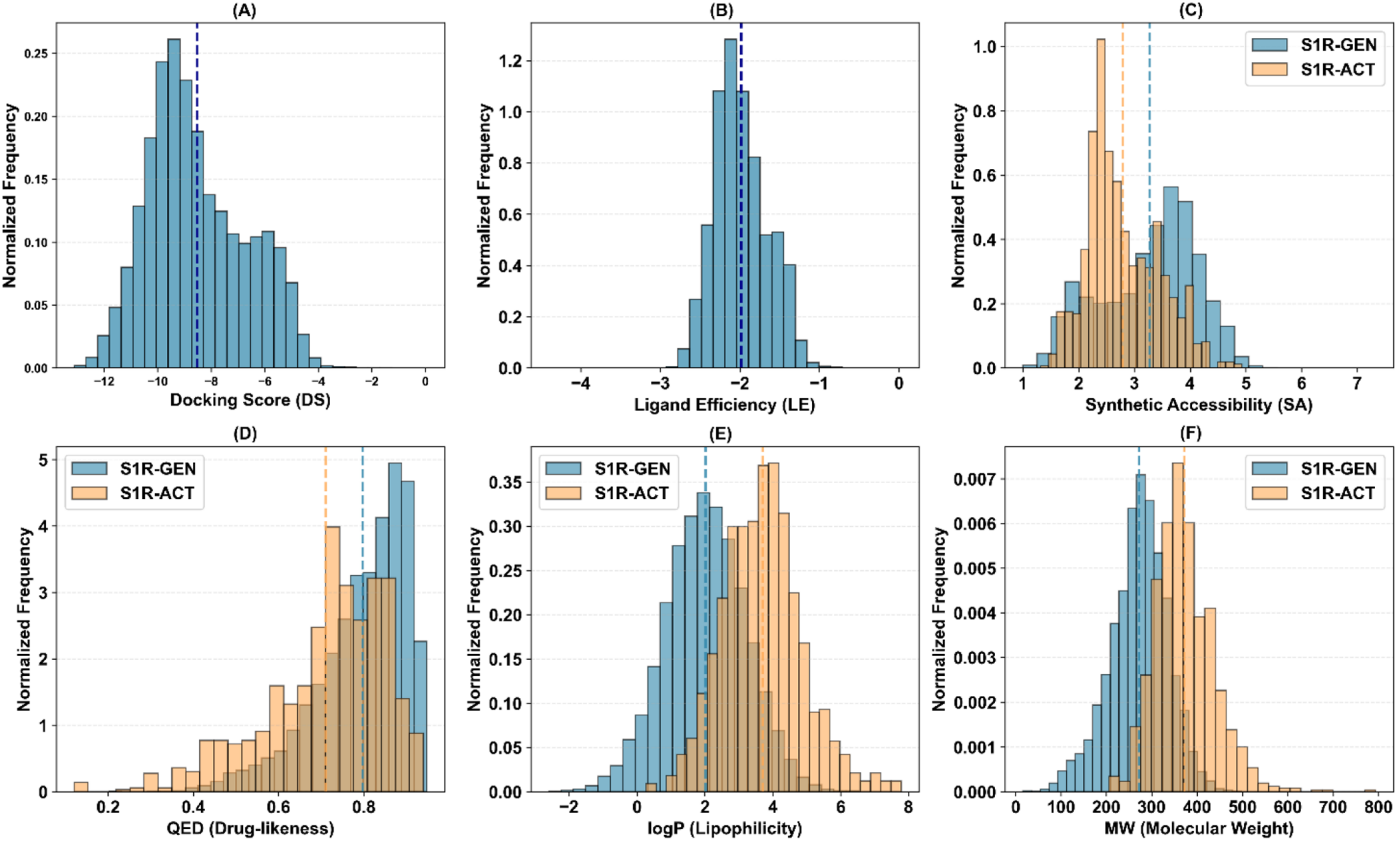
Property distributions for the S1R hit identification campaign: A) docking score (DS), B) ligand efficiency (LE), C) synthetic accessibility (SA), D) quantitative estimate of drug-likeness (QED), E) lipophilicity (logP), and F) molecular weight (MW). Distributions for generated compounds (S1R–GEN) and known actives found in ChEMBL v36 (S1R–ACT) are colored in blue and orange, respectively. Mean values are reported via vertical dashed lines.

**Figure 5:**
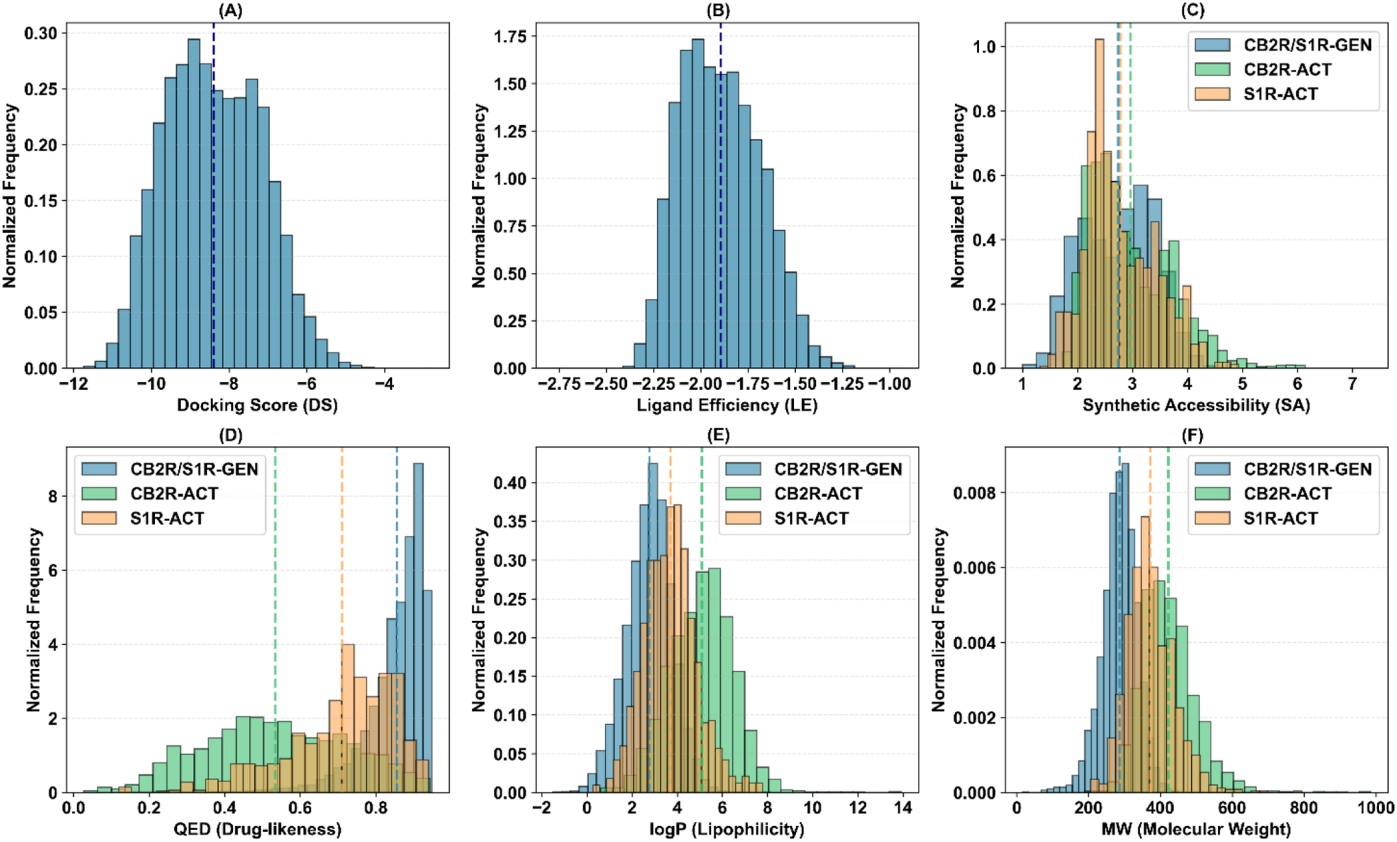
Property distributions for the CB2R/S1R dual target hit identification campaign: A) docking score (DS), B) ligand efficiency (LE), C) synthetic accessibility (SA), D) quantitative estimate of drug-likeness (QED), E) lipophilicity (logP), and F) molecular weight (MW). Distributions for generated compounds (CB2R–GEN) and known actives found in ChEMBL v36 (CB2R–ACT and S1R–ACT) are colored in blue, green, and orange, respectively. Mean values are reported via vertical dashed lines.

**Table 2:** Comprehensive Quality Metrics of Generated Hit Identification Libraries across Three Optimization Scenarios^a^

Metric	CB2R-GEN	CB2R-ACT	S1R-GEN	S1R-ACT	CB2R/S1R-GEN
**DS**	-8.99 ± 1.14	N/A	-8.53 ± 1.83	N/A	-8.40 ± 1.23
**LE**	-2.03 ± 0.20	N/A	-1.98 ± 0.33	N/A	-1.89 ± 0.21
**SA**	2.30 ± 0.50	2.97 ± 0.75	3.27 ± 0.86	2.79 ± 0.64	2.74 ± 0.67
**QED**	0.84 ± 0.09	0.53 ± 0.19	0.80 ± 0.11	0.71 ± 0.14	0.86 ± 0.08
**logP**	3.10 ± 0.95	5.09 ± 1.40	2.02 ± 1.18	3.70 ± 1.16	2.77 ± 1.01
**MW**	290.42 ± 47.55	422.75 ± 74.82	271.81 ± 63.74	372.11 ± 65.28	286.01 ± 48.53
**ID**	0.82	0.85	0.87	0.84	0.85
**T**_**MAX**_	0.29 ± 0.05	N/A	0.24 ± 0.06	N/A	0.29 ± 0.05
**S**_**d**_	0.06	N/A	0.25	N/A	0.17

a For each campaign (CB2R, S1R, dual CB2R/S1R), data report docking mean score (DS), ligand efficiency (LE), synthetic accessibility (SA),^16^ quantitative estimate of drug-likeness (QED),^27^ lipophilicity (logP), molecular weight (MW), internal diversity (ID), maximum Tanimoto similarity relative to known actives (T_MAX_), and scaffold diversity (S_d_). Quality metrics for known active molecules (CB2R-ACT, S1R-ACT) found in ChEMBL version 36 are reported for comparison.

For CB2R, optimization proceeded through a steep initial improvement in DS, followed by smooth stabilization of both the best and average scores (Figure 2A), indicating rapid identification of a narrow set of highly favorable pharmacophores. In contrast, the S1R campaign displayed a more protracted and volatile trajectory (Figure 2B), characterized by repeated cycles of discovery and replacement of elite scaffolds before eventual convergence, consistent with the receptor’s well-known ligand promiscuity.^51^ The dual-target CB2R/S1R experiment displayed intermediate behavior (Figure 2C), converging more slowly than CB2R-based generation but with lower volatility compared to the generation performed to target S1R only. This trend reflects the additional constraint introduced by the harmonic mean objective, which systematically penalizes single-target specialists while favoring balanced polypharmacological profiles.

The CB2R hit identification run produced a chemically diverse library of 12,319 unique molecules (Figure 3), tightly clustered around highly favorable DS and excellent LE values. The mean DS approached −8.99 ± 1.14 kcal/mol, with the best candidates around −12 kcal/mol, while maintaining an average LE of −2.03 ± 0.20 kcal/mol. Strikingly, these gains in predicted binding affinity were not achieved at the expense of molecular simplicity: the average synthetic accessibility (SA) remained at 2.30 ± 0.50, and the quantitative estimate of drug-likeness (QED) was centered around 0.84 ± 0.09 with narrow dispersion, indicating a consistently drug-like output distribution. The RL-guided GA preferentially generated comparatively lean chemotypes with an average molecular weight (MW) of 290.42 ± 47.55 Da and a mean logP of 3.10 ± 0.95. Known human CB2R binders (source CHEMBL v36 – ID: CHEMBL 253) tend, on average, to display higher MW (422.75 ± 74.82 Da) and lipophilicity (logP = 5.09 ± 1.40), suggesting a relative enrichment of smaller and less lipophilic structures in the generated set. This shift toward lower MW and moderate lipophilicity, while preserving or improving predicted affinity, highlights the capacity of the framework to identify high-novelty hits potentially mitigating liabilities related to solubility.

It is worth noting that the generated molecules do not have analogues among those known to be active on CB2R, as indicated by the low average maximum Tanimoto similarity value of the generated molecules with respect to the known active compounds (T_MAX_ = 0.29 ± 0.05, calculated using Morgan circular fingerprints with radius 2), thus highlighting their high novelty. A representative set of three molecules is shown in Figure 6 while the SMILES strings of the entire set of generated compounds are available as Supporting Information. Notice that all of the compounds displayed in Figure 6 were not selected exclusively based on their ranking scores, as top-ranked molecules are often structurally similar. Instead, they were chosen to illustrate a user-driven selection process that also considers diversity, novelty, and practical constraints. The three structures display no similarity to compounds known to be active on CB2R (T_MAX_ values of 0.29, 0.26, and 0.25 for IDs 5213, 11560, and 11797, respectively) and share a common moiety characterized by two phenyl rings directly linked to each other. This scaffold has previously been identified in active CB2R ligands,^61–63^ indicating ALCHIMIA’s capability to rediscover known pharmacophoric motifs. Although this observation requires experimental validation to be confirmed, the generated molecules themselves have no previously reported biological activity and represent novel chemical entities, as indicated by a PubChem database search.^64^ To provide a more concrete understanding of how the algorithm operates, the pathways that led, through the selection of different actions, to the generation of the molecules shown in the figure are reported in the Supporting Information (Figures S2–S4).

**Figure 6:**
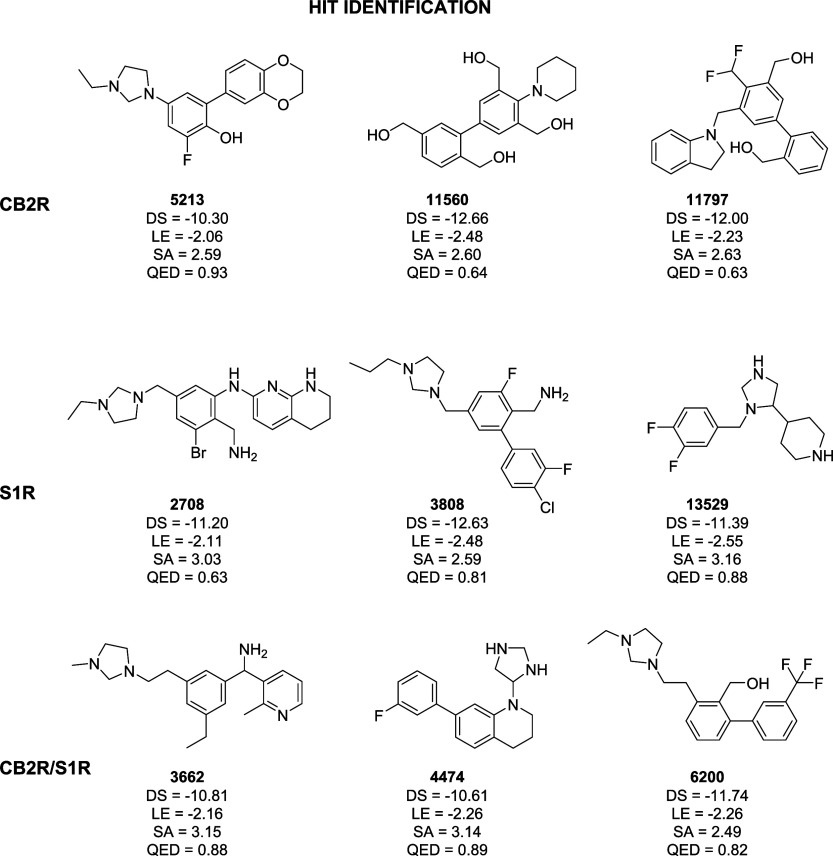
Compounds selected from single-target CB2R and S1R and dual-target CB2R/S1R hit identification generation campaigns, along with computed docking score (DS in kcal/mol), ligand efficiency (LE in kcal/mol), and QED and SA values.

As far as the generation of S1R hit candidates is concerned, ALCHIMIA generated 20,719 unique molecules (Figure 4), but the optimization landscape proved markedly more rugged. The mean DS improved more gradually, attaining −8.53 ± 1.83 kcal/mol, and exhibited broader fluctuations, while the global best continued to be replaced over an extended generational window, indicating the presence of multiple local optima corresponding to structurally distinct solutions. This behavior is fully consistent with the pharmacological promiscuity of S1R, which accommodates chemically diverse scaffolds such as benzomorphans, tricyclic antidepressants, and phenothiazines.^51^ The final S1R set converged toward ligands with LEs close to −1.98 ± 0.33 kcal/mol (Figure 4). The RL policy continued to enforce favorable property profiles: SA reached an average value of 3.27 ± 0.86; an average QED value of 0.80 ± 0.11 was obtained, and the library was systematically biased toward lower molecular weight (271.81 ± 63.74 Da) and significantly reduced lipophilicity compared to known S1R actives. This lipophilicity drop is especially relevant for S1R, where excessive lipophilicity has been linked to problematic off-target pharmacology at opioid receptors, monoamine transporters, and other central nervous system targets.^65^ Notably, the generated molecules differ from those previously reported for S1R (T_MAX_ = 0.24 ± 0.06) but, compared to the CB2R case, exhibit a higher degree of scaffold diversity (S_d_), defined as the ratio between the number of unique Bemis–Murcko scaffolds^66^ found in the generated set of molecules and the size of the set (0.25 vs 0.06), consistent with the higher S1R promiscuity. Figure 6 shows a representative set, while their docking poses and the complete list of generated compounds are provided in the Supporting Information (Figure S17). The three molecules, all characterized by a high degree of novelty with respect to known S1R binders (T_MAX_ equal to 0.24, 0.33, and 0.33 for IDs 2708, 3808, and 13529, respectively), display substantial structural diversity while retaining key features known to be crucial for binding with the receptor, such as the presence of at least one protonatable nitrogen atom, which is essential for the interaction with E172.^67^ The pathways that led to the generation of the molecules shown in the figure are reported in the Supporting Information (Figures S5–S7). Importantly, the analysis of the obtained poses allows the identification of key interactions commonly exhibited by ligands of these receptors. First, the three S1R candidates feature a positively ionizable nitrogen atom that forms a salt bridge, reinforced by a hydrogen bond, with Glu172. This interaction represents a distinctive requirement for all S1R ligands.^68^ As far as CB2R is concerned, the proposed binding modes of the three selected compounds display commonly encountered interactions observed in multiple experimentally solved receptor–ligand complexes, specifically the typical π–π interactions between the ligands and residues Phe87, Phe94, and Phe183, as well as the hydrogen bond with the side chain Thr114 or the backbone of Leu182.^50,69–71^

It is also worth noting that generating molecules starting from CH_4_—thus without any prior knowledge of other ligands capable of binding the receptors—enables the exploration of novel chemical space without compromising the drug-likeness of the resulting compounds. This is further supported by the Uniform Manifold Approximation and Projection (UMAP) analysis,^72^ based on 36 physicochemical and topological properties of the molecules, calculated using the RDKit Descriptor Calculation node within the KNIME platform,^73^ in which we compared the chemical space spanned by the CB2R and S1R libraries with a library comprising approved drugs downloaded from ChEMBL v36. This approach enables visual exploration of the distribution of the compounds in a two-dimensional chemical space. The resulting plots (Figure S18) show substantial overlap, suggesting that the generated data sets effectively cover relevant regions of drug-like chemical space, thus supporting the quality of the molecules generated by ALCHIMIA.

Finally, to further characterize the generated libraries, we performed an activity cliff analysis on the CB2R and S1R hit identification sets by identifying molecule pairs with Tanimoto similarity ≥0.8 (Morgan Fingerprints, radius 2) and a docking score difference ≥2 kcal/mol. This analysis revealed target-specific patterns in the transformations most frequently associated with significant docking score variations (Figure S19). For S1R, potential activity cliffs are driven by a narrower set of modifications, such as polarity swaps and ring contractions, while for CB2R, a more distributed profile was observed, dominated by bioisosteric replacements and chain length modulations. These findings provide useful indications regarding which structural modifications may be advantageous or detrimental for binding to each receptor, further underscoring the interpretability of ALCHIMIA’s transformation vocabulary.

The dual CB2R/S1R hit identification scenario represents the most stringent test of the framework, as ALCHIMIA was required to optimize a harmonic mean fitness function:

$$F=2\cdot LE_{1}\cdot LE_{2}/\left(LE_{1}+LE_{2}\right)$$

where *LE*_1_ and *LE*_2_ correspond to the ligand efficiency returned after docking the considered molecule on the first and second target, respectively. Such a function was employed to favor molecules with balanced affinity against both targets (*i.e.;* S1R and CB2R), hence penalizing single-target specialists (Figure 2). Under this polypharmacological objective, the algorithm generated a library of 12,166 unique compounds (Figure 5). The resulting hit candidates exhibited mean harmonic DS equal to −8.40 ± 1.23 kcal/mol with LEs of −1.89 ± 0.21 kcal/mol (Figure 5), thus only marginally inferior to the single-target CB2R campaign and comparable to the S1R campaign, thereby demonstrating that balanced dual-target binding can be achieved without substantial sacrifice in per-atom binding efficiency. Notably, this performance was accompanied by favorable physicochemical profiles (SA = 2.74 ± 0.67; QED = 0.86 ± 0.08), together with relatively low MW (286.01 ± 48.53 Da) and logP (2.77 ± 1.01) values. Overall, these results suggest that the multitarget ligand design strategy adopted here does not inherently require increased molecular size and lipophilicity, in contrast to trends commonly associated with pharmacophore linking or merging approaches.^17^ In other words, the RL-guided GA preferentially identifies compact, fused scaffolds that exploit overlapping or closely positioned pharmacophoric features to simultaneously engage both CB2R and S1R. This architectural convergence preserves high LE and favorable drug-likeness while potentially enabling balanced dual-target activity,^17^ a consideration that warrants further investigation for subsequent experimental validation.

A representative set of dual candidates is shown in Figure 6 (generation pathways reported in Figures S8–S10 of the Supporting Information). Interestingly, all molecules display the key feature of S1R ligands (i.e., the presence of a protonatable nitrogen atom). Compounds 4474 and 6200 also reproduce the scaffold consisting of two linked phenyl rings observed in the dataset of CB2R hit candidates. In other words, ALCHIMIA appears capable of preserving the structural features required to engage both receptors, as expected for a dual-oriented generator. Importantly, these compounds exhibit a high degree of structural novelty. No entries corresponding to these molecules are currently available in PubChem,^64^ and scaffolds of this kind have not previously been explored as CB2R/S1R dual modulators.

#### Application to Lead Optimization

To assess ALCHIMIA’s suitability for lead optimization tasks, additional optimization tasks and additional generations were performed starting from the scaffold of known active compounds and constraining all generated molecules to retain the corresponding core structure. For CB2R, we selected JWH133 (dimethylbutyldeoxy-Δ^8^-THC) , a well-known selective CB2R agonist.^74^ For S1R, the chosen reference ligand was (+)-pentazocine, a high-affinity and well-characterized S1R agonist.^75^ Notably, both compounds, whose 2D structures are shown in Figure 7, exhibit low nanomolar binding affinities toward their respective targets.^74,75^ During the generation, the considered scaffold (Figure 7) was constrained to remain unchanged, while all other portions of the structure were allowed to vary freely. This setup was designed to closely mimic a typical lead-optimization scenario. As for the hit identification tasks, sim_cut_ was fixed at 0.4.

**Figure 7:**
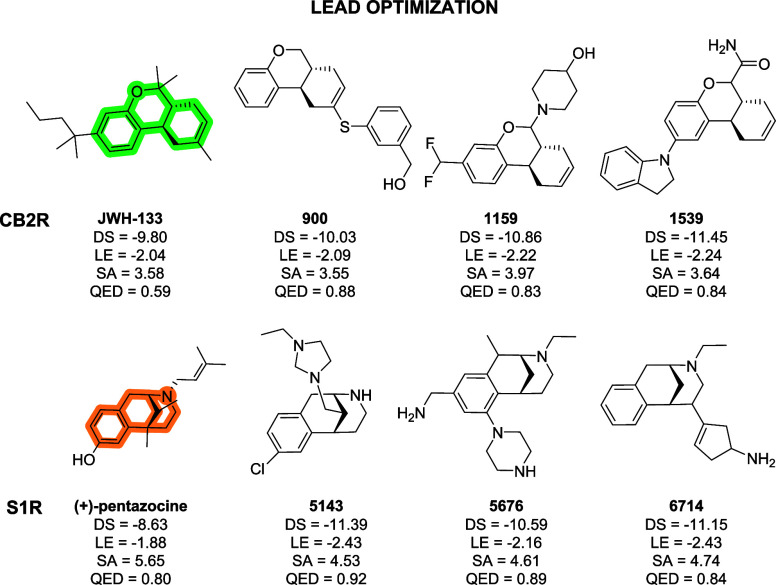
JWH-133 and (+)-pentazocine structures and compounds selected from CB2R and S1R lead optimization generation campaigns, along with computed docking score (DS in kcal/mol), ligand efficiency (LE in kcal/mol), and QED and SA values. The scaffolds used as constraints for lead optimization generation campaigns are highlighted in green and orange for JWH-133 and (+)-pentazocine, respectively.

As for JWH-133, ALCHIMIA reached convergence using the same criteria described above (convergence statistics reported in Figure S1A of the Supporting Information), ultimately generating a library of 6,726 compounds derived from distinct optimization hypotheses. As in the previous sections, three selected examples are presented here (Figure 7, generation pathways reported in Figures S11–S13 of Supporting Information), while the full data set is provided in the Supporting Information. Notably, all three compounds feature scaffold substitutions alternative to JWH-133, resulting in improved DS, LE, and QED compared to the known active binder (DS = −9.80 kcal/mol; LE = −2.04; QED = 0.59) while maintaining comparable synthetic accessibility (SA for JWH-133 = 3.58). A similar analysis can be performed on the compounds generated from the pentazocine scaffold (library of 9,570 compounds; convergence statistics reported in Figure S1B of the Supporting Information). Again, considering, for example, the three selected representative compounds (Figure 7, generation pathways reported in Figures S14–S16 of the Supporting Information), a notable diversity in scaffold substitutions is observed, leading to distinct optimization hypotheses that result in significant improvements over pentazocine in terms of DS, LE, and SA, while maintaining a comparable QED value (QED = 0.80 for pentazocine). In summary, these generations suggest that ALCHIMIA can serve as an effective algorithm to guide the optimization of a lead compound or to provide alternative optimization pathways beyond those previously explored.

### Comparison with State-of-The-Art Methods

Benchmarking in *de novo* molecular design is typically anchored to two frameworks, namely MOSES and GuacaMol, which together set expectations for what a modern generator should deliver, providing validity, uniqueness, novelty, and property-distribution metrics.^12,76^

In particular, we considered the Novelty, Scaffold Novelty, Fragment Similarity, Scaffold Similarity, Fréchet ChemNet Distance (FCD), and KL Divergence metrics fully described in these works. These metrics were computed on the molecules generated by ALCHIMIA’s RL policy starting from the ChEMBL test set compounds for the values of M employed in the GA applications (M = 1, 2, 3), as reported in Table 3. Remarkably, ALCHIMIA generates molecules that are novel (high Novelty and Scaffold Novelty) yet chemically realistic, maintaining close alignment with the parent distribution in terms of fragments, scaffolds, and physicochemical property distributions (high Scaffold and Fragment Similarity, low FCD, KL Divergence close to 1), while simultaneously achieving high drug-likeness and synthetic accessibility through RL-guided optimization. Moreover, the Scaffold Novelty as well as the FCD increase from M = 1 to M = 3. This indicates that the model can generate novel scaffolds and fragments, allowing an incremental exploration of the chemical space. Furthermore, this analysis enables a direct comparison with established generative models.^12,76^ Specifically, we can compare ALCHIMIA’s performance against the Adversarial Autoencoder (AAE),^78,79^ Variational Autoencoder (VAE),^80^ CharRNN,^81^ Junction Tree Variational Autoencoder (JTN-VAE),^82^ LatentGAN,^83^ SMILES LSTM,^76^ and ORGAN^84^ as reported in both MOSES and GuacaMol. The results reveal that ALCHIMIA achieves distribution-learning performance on par with AAE (Novelty = 0.998, FCD = 1.057, KL Divergence = 0.886), as well as with VAE and JTN-VAE (Novelty = 0.974, FCD = 0.938, KL Divergence = 0.982).

**Table 3:** Distribution-Learning Metrics Computed on Molecules Generated by ALCHIMIA’s RL Policy from the ChEMBL Test Set at Trajectory Lengths M = 1, 2, and 3, following the Evaluation Frameworks Proposed by GuacaMol^76^ and MOSES^a^ ^12^

Metric	M = 1	M = 2	M = 3
**Novelty**	0.95	0.94	0.95
**Scaffold Novelty**	0.27	0.48	0.57
**Fragment Similarity**	0.99	0.98	0.97
**Scaffold Similarity**	0.98	0.95	0.93
**FCD**	0.44	1.02	1.37
**KL Divergence**	0.87	0.84	0.82

a Novelty: fraction of generated molecules not present among the parent test set molecules. Scaffold Novelty: fraction of Bemis–Murcko scaffolds in the generated set that are absent from the parent test set. Fragment Similarity and Scaffold Similarity: cosine similarity between the frequency distributions of BRICS fragments^30^ and Bemis–Murcko scaffolds,^66^ respectively, of the generated molecules and the parent test set molecules. Fréchet ChemNet Distance (FCD):^77^ distance between the penultimate-layer ChemNet activations of the generated and parent sets. KL Divergence: average Kullback–Leibler divergence computed over the distributions of nine physicochemical descriptors (BertzCT, MolLogP, MolWt, TPSA, NumHAcceptors, NumHDonors, NumRotatableBonds, NumAliphaticRings, and NumAromaticRings) between the generated and parent molecules. The interested reader is referred to GuacaMol^76^ and MOSES^12^ for additional computational details.

Regarding the GA results, within this context, the quantitative properties of ALCHIMIA’s libraries are on par with leading benchmark results, as supported by MW and logP values returned by the generations performed in the hit identification scenario. Moreover, ALCHIMIA maintains consistently high QED values (CB2R = 0.84 ± 0.09; S1R = 0.80 ± 0.11; CB2R/S1R = 0.86 ± 0.08) and SA scores in the easy-to-moderate synthesizability range (CB2R = 2.30 ± 0.50; S1R = 3.27 ± 0.86; CB2R/S1R = 2.74 ± 0.67). These distributions echo the MOSES design principles of compactness and moderated lipophilicity and demonstrate that explicit optimization of QED and SA produces libraries that need minimal *posthoc* pruning, addressing a common shortcoming of pure distribution learning approaches. ALCHIMIA shares the reinforcement learning philosophy of recent frameworks such as REINVENT^85^ and ACEGEN^14^ but departs in two practical respects that improve *in-silico*-to-bench practicality.

First, it replaces token-level sequence actions with an interpretable vocabulary of 33 medicinal chemistry transformations, so each policy action corresponds to a clear chemical edit (functional group modifications, ring operations, bioisosteric swaps, chain tuning). This approach follows fragment/context-aware mutation frameworks such as CReM^24^ while extending them by learning which transformations to prioritize under explicit QED/SA rewards.

Second, ALCHIMIA couples the learned policy to a docking-guided GA that enforces population diversity and selects elites by LE, thereby integrating physics-aware scoring directly into the optimization loop rather than relying solely on property proxies. On the evolutionary and guided-mutation front, methods such as CReM,^24^ DockingGA,^22^ and GENERA^21^ illustrate the value of domain-aware operators combined with GA search: CReM ensures RDKit-valid structures and exposes controls over the context radii and fragment libraries. At the same time, DockingGA and GENERA show that simple, well-chosen operators, combined with physics-based fitness, can match or outperform more complex neural pipelines on targeted objectives. ALCHIMIA aligns with this family but adds a learned ranking: its mutational vocabulary remains chemically interpretable (avoiding latent token insertions), and mutations are prioritized by a trained policy that internalizes trade-offs among SA, QED, and structural drift. The outcome is compact libraries (lower MW and logP) that remain competitive in predicted affinity, an empirical pattern consistently observed in our CB2R and S1R campaigns. Finally, unlike other *de novo* design methods that require at least a pool of known actives as starting points (e.g., GENERA),^21^ ALCHIMIA enables target-oriented generation even in the complete absence of known binders. Indeed, the runs reported here (Figure 7) were initiated from methane as the sole seed.

### Overall Comments and Conclusions

This work presents a fundamentally novel hybrid computational framework that synergistically integrates RL-guided GAs with medicinal chemistry-inspired molecular transformation operations, establishing an interpretable and mechanistically transparent approach to *de novo* drug discovery that directly addresses critical limitations of contemporary generative models.^4^ The principal innovation is the replacement of implicit latent-space operations with a chemically interpretable vocabulary of 33 medicinal-chemistry transformations. By using these explicit operators rather than latent token manipulations, ALCHIMIA departs from purely data-driven deep generative architectures and yields a generative model that is substantially more transparent and actionable for medicinal chemists. The mechanistic advantages of this hybrid approach are substantial and multifaceted. Primarily, the SA evaluation is fundamentally integrated into the generation process rather than applied as a *posthoc* filter, ensuring that all generated molecules lie within a synthetically accessible chemical space by construction. This design principle directly addresses a critical limitation of contemporary deep generative models, where molecules achieving impressive *in silico* property predictions frequently prove to be synthetically intractable upon experimental synthesis attempts. Furthermore, the framework simultaneously optimizes multiple conflicting objectives, such as binding affinity, synthetic feasibility, drug-likeness, lipophilicity, and molecular weight, through balanced multicriteria fitness evaluation. Finally, the explicit encoding of chemical operations ensures that all generated molecules remain valid, chemically reasonable structures, eliminating the typical problem where naive generative models produce substantial fractions of chemically invalid or unfeasible structures requiring downstream filtering and validation. ALCHIMIA was assessed in five distinct drug discovery scenarios across two therapeutically relevant receptors (CB2R and S1R), including unconstrained hit discovery, dual target polypharmacology optimization, and scaffold constrained lead optimization. This multifaceted evaluation permitted a direct comparison of competing objectives, such as novelty driven hit discovery versus scaffold preserving lead optimization, and of target complexity, from single target to dual target constraints, thereby mirroring the practical challenges of medicinal chemistry. Compounds generated by ALCHIMIA exhibit high structural novelty relative to known actives and consistently outperform in terms of SA and QED scores, while also showing promising predicted binding affinities. Furthermore, generating molecules starting from CH_4_—thus without relying on prior knowledge of compounds capable of binding the target—reduces the risk of bias and enables broader exploration of chemical space, potentially increasing the likelihood of overcoming existing patent barriers and expanding the patentable landscape. From a practical standpoint, ALCHIMIA offers clear advantages over purely data-driven methods. By encoding explicit medicinal-chemistry transformations as the generative vocabulary, every proposed modification is accompanied by a transparent chemical rationale, so the algorithm’s decisions are interpretable and actionable. Each generated molecule can be traced back to a sequence of discrete transformation steps, allowing medicinal chemists to assess whether suggested edits conform to target-specific structure–activity relationships (SAR) and synthetic constraints. This mechanistic transparency contrasts sharply with black-box deep-learning generators that operate in latent space and provide little or no explicable design rationale, thereby facilitating more informed prioritization and faster bench-side translation. Additionally, ALCHIMIA is readily applicable to both hit discovery/optimization and lead optimization workflows. In hit-oriented use cases, no known binders are required to generate candidate molecules, making it the first method, to the best of our knowledge, capable of generating focused libraries directly from the protein’s 3D structure—an information which is increasingly available through modern experimental techniques (e.g., cryo-EM) and AI-based predictors such as AlphaFold.^86^ It is important to note that, although docking approaches are widely used in virtual screening, their scoring functions have limited reliability in quantitatively predicting binding affinities.^87^ Accordingly, in this work, DS are treated as heuristic ranking signals rather than definitive predictors, as docking represents only one of the possible predictive approaches that can be integrated within ALCHIMIA. The framework is designed to flexibly accommodate multiple predictive methods, enabling future applications to incorporate multitool scoring schemes or cross-docking validation. Thus, docking is included here primarily as a proof-of-concept to illustrate how ALCHIMIA can integrate external predictive models rather than implying a direct or accurate correlation with experimental binding data. By codifying medicinal chemistry intuition into learnable transformation operations while leveraging RL capacity for multiobjective optimization and genetic algorithms’ population-based diversity maintenance, ALCHIMIA represents a practical and interpretable approach to computational drug design that augments rather than replaces human expertise. This transparent, chemically grounded framework addresses the critical need for user-friendly tools in medicinal chemistry industrial workflows, where transparency, chemical plausibility, and alignment with practitioner expertise remain paramount considerations.^65^

## Supplementary Material













## Data Availability

The code of the developed RL and GA algorithms is freely available as a GitHub repository at https://github.com/alberdom88/ALCHIMIA.

## Notes

Figure S1: Genetic algorithm convergence profiles for lead optimization campaigns: A) CB2R and B) S1R; Figure S2–S10: Genetic algorithm pathway that led to the generation of compounds in Figure 6; Figures S11–S16: Genetic algorithm pathway that led to the generation of compounds in Figure 7; Figure S17: Docking poses of representative set of generated compound in the CB2R (A) and S1R (B) hit identification campaign; Figure S18: Chemical space visualization by Uniform Manifold Approximation and Projection (UMAP) of CB2R (A) and S1R (B) compounds from the hit identification campaign versus approved drugs retrieved from ChEMBL; and Figure S19: Frequency of molecular transformations associated with potential activity cliff pairs in the CB2R (green) and S1R (orange) hit identification libraries (DOCX)
cb2r_hit_identification.csv: Compounds generated through the CB2R hit identification campaign (CSV)
s1r_hit_identification.csv: Compounds generated through the S1R hit identification campaign (CSV)
cb2r_s1r_dual_hit_identification.csv: Compounds generated through the CB2R/S1R dual target hit identification campaign (CSV)
cb2r_lead_optimization.csv: Compounds generated through the CB2R lead optimization campaign (CSV)
s1r_lead_optimization.csv: Compounds generated through the S1R lead optimization campaign (CSV)
