# Supplementary material for: A Reinforcement Learning-Guided Genetic Algorithm Integrating Medicinal Chemistry-Inspired Molecular Transformations
Source: J Chem Inf Model. 2026 Apr 16;66(8):4576–91. doi: 10.1021/acs.jcim.6c00397 (PMC13126637; doi:10.1021/acs.jcim.6c00397)
Supplement: Supplementary file 1 [file ci6c00397_si_001.docx]

**A Reinforcement Learning-guided Genetic Algorithm Integrating Medicinal Chemistry-Inspired Molecular Transformations**

*Domenico Alberga,^a^* Vittoria Nanna,^a^ Maria Giovanna Eva Papadopoulos,^a^ Adolfo Ancona,^a,b^ Maria Cristina Lomuscio,^c^ and Giuseppe Felice Mangiatordi^a^**

^a^ CNR – Institute of Crystallography, Via Amendola 122/o, 70126 Bari, Italy.

^b^ Department of Biotechnology, Chemistry and Pharmacy, University of Siena, 53100, Siena, Italy.

^c^ Dipartimento di Medicina di Precisione e Rigenerativa e Area Jonica (DiMePRe-J), Università degli Studi di Bari Aldo Moro, Piazza Giulio Cesare, 11, Policlinico, 70124, Bari, Italy.

TABLE OF CONTENTS

**Figure S1.** Genetic algorithm convergence profiles for lead optimization campaigns: A) CB2R B) S1R. For each generation, the left panels show the best and mean docking scores of the evolving population as purple and blue lines, respectively. Yellow diamonds indicate generations where a new global minimum is first observed, while green squares indicate generations where a new best mean score is obtained. The right panels report the population size per generation, with red triangles marking generations in which the population drops below the threshold of 100, highlighted by a horizontal red dashed line.

**Figure S2.** Genetic algorithm pathway that led to the generation of compound 5213 of CB2R hit identification campaign. For each step docking score (DS), quantitative estimate of drug-likeness (QED) and synthetic accessibility (SA) scores are reported.

**Figure S3.** Genetic algorithm pathway that led to the generation of compound 11560 of CB2R hit identification campaign. For each step docking score (DS), quantitative estimate of drug-likeness (QED) and synthetic accessibility (SA) scores are reported.

**Figure S4.** Genetic algorithm pathway that led to the generation of compound 11797 of CB2R hit identification campaign. For each step docking score (DS), quantitative estimate of drug-likeness (QED) and synthetic accessibility (SA) scores are reported.

**Figure S5.** Genetic algorithm pathway that led to the generation of compound 2708 of S1R hit identification campaign. For each step docking score (DS), quantitative estimate of drug-likeness (QED) and synthetic accessibility (SA) scores are reported.

**Figure S6.** Genetic algorithm pathway that led to the generation of compound 3808 of S1R hit identification campaign. For each step docking score (DS), quantitative estimate of drug-likeness (QED) and synthetic accessibility (SA) scores are reported.

**Figure S7.** Genetic algorithm pathway that led to the generation of compound 13529 of S1R hit identification campaign. For each step docking score (DS), quantitative estimate of drug-likeness (QED) and synthetic accessibility (SA) scores are reported.

**Figure S8.** Genetic algorithm pathway that led to the generation of compound 3662 of CB2R/S1R dual target hit identification campaign. For each step docking score (DS), quantitative estimate of drug-likeness (QED) and synthetic accessibility (SA) scores are reported.

**Figure S9.** Genetic algorithm pathway that led to the generation of compound 4474 of CB2R/S1R dual target hit identification campaign. For each step docking score (DS), quantitative estimate of drug-likeness (QED) and synthetic accessibility (SA) scores are reported.

**Figure S10.** Genetic algorithm pathway that led to the generation of compound 6200 of CB2R/S1R dual target hit identification campaign. For each step docking score (DS), quantitative estimate of drug-likeness (QED) and synthetic accessibility (SA) scores are reported.

**Figure S11.** Genetic algorithm pathway that led to the generation of compound 900 of CB2R lead optimization campaign. For each step docking score (DS), quantitative estimate of drug-likeness (QED) and synthetic accessibility (SA) scores are reported.

**Figure S12.** Genetic algorithm pathway that led to the generation of compound 1159 of CB2R lead optimization campaign. For each step docking score (DS), quantitative estimate of drug-likeness (QED) and synthetic accessibility (SA) scores are reported.

**Figure S13.** Genetic algorithm pathway that led to the generation of compound 1539 of CB2R lead optimization campaign. For each step docking score (DS), quantitative estimate of drug-likeness (QED) and synthetic accessibility (SA) scores are reported.

**Figure S14.** Genetic algorithm pathway that led to the generation of compound 5143 of S1R lead optimization campaign. For each step docking score (DS), quantitative estimate of drug-likeness (QED) and synthetic accessibility (SA) scores are reported.

**Figure S15.** Genetic algorithm pathway that led to the generation of compound 5676 of S1R lead optimization campaign. For each step docking score (DS), quantitative estimate of drug-likeness (QED) and synthetic accessibility (SA) scores are reported.

**Figure S16.** Genetic algorithm pathway that led to the generation of compound 6714 of S1R lead optimization campaign. For each step docking score (DS), quantitative estimate of drug-likeness (QED) and synthetic accessibility (SA) scores are reported.

**Figure S17.** Docking poses of representative set of generated compounds in the CB2R (A) and S1R (B) hit identification campaign.

**Figure S18.** Chemical space visualization by Uniform Manifold Approximation and Projection (UMAP) of CB2R (A) and S1R (B) compounds from the hit identification campaign versus approved drugs retrieved from ChEMBL, based on 36 physicochemical and topological descriptors calculated using RDKit Descriptor Calculation node within the KNIME platform.

**Figure S19**. Frequency of molecular transformations associated with potential activity cliff pairs in the CB2R (green) and S1R (orange) hit identification libraries. Activity cliff pairs were defined as molecule pairs with Tanimoto similarity ≥ 0.8 (Morgan Fingerprints, radius 2) and a docking score difference ≥ 2 kcal/mol.

**Figure S1.**

**
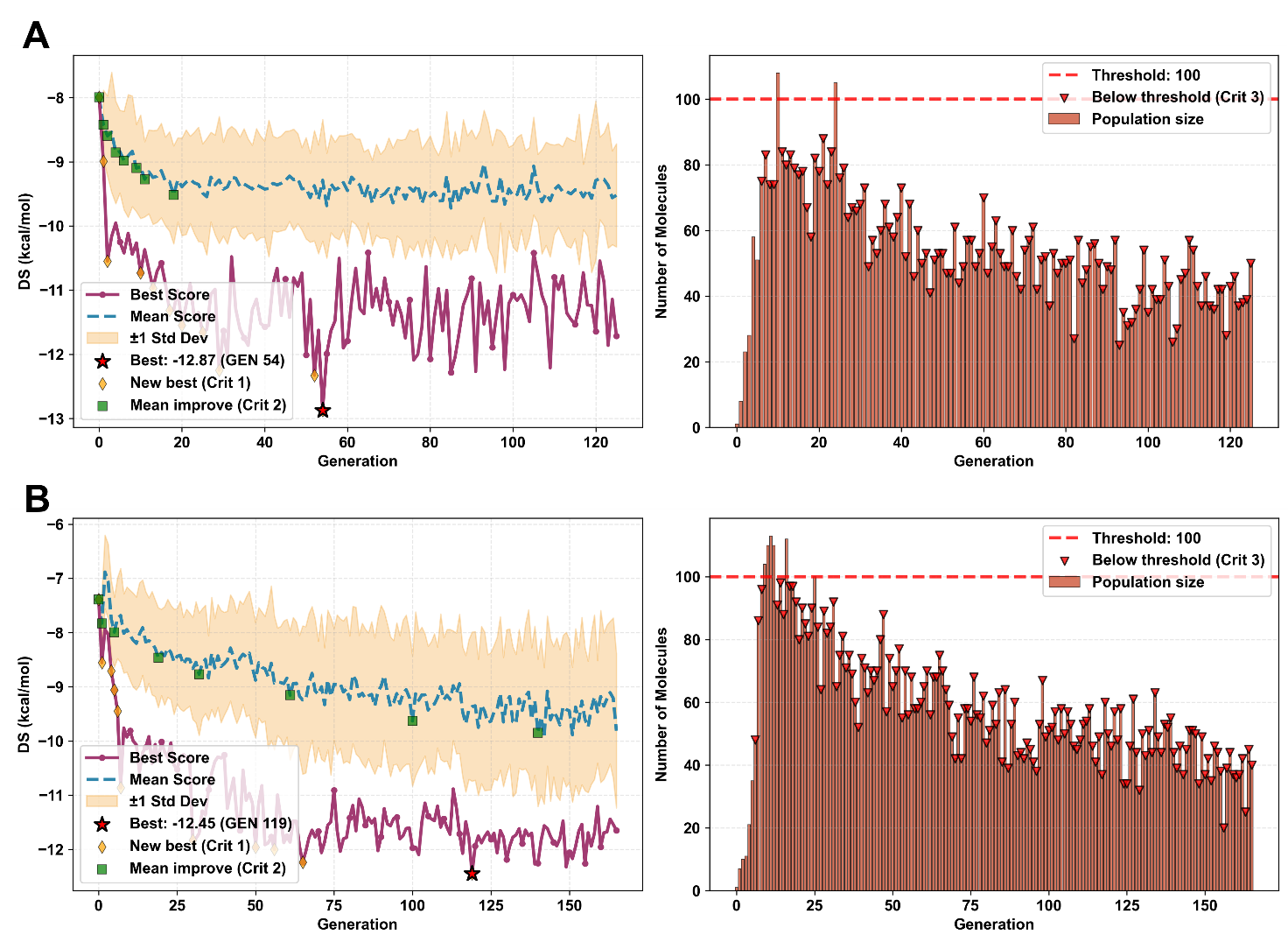
**

**Figure S2.**

**Figure S3.**

**Figure S4.**

**Figure S5.**

**Figure S6.**

**Figure S7.**

**Figure S8.**


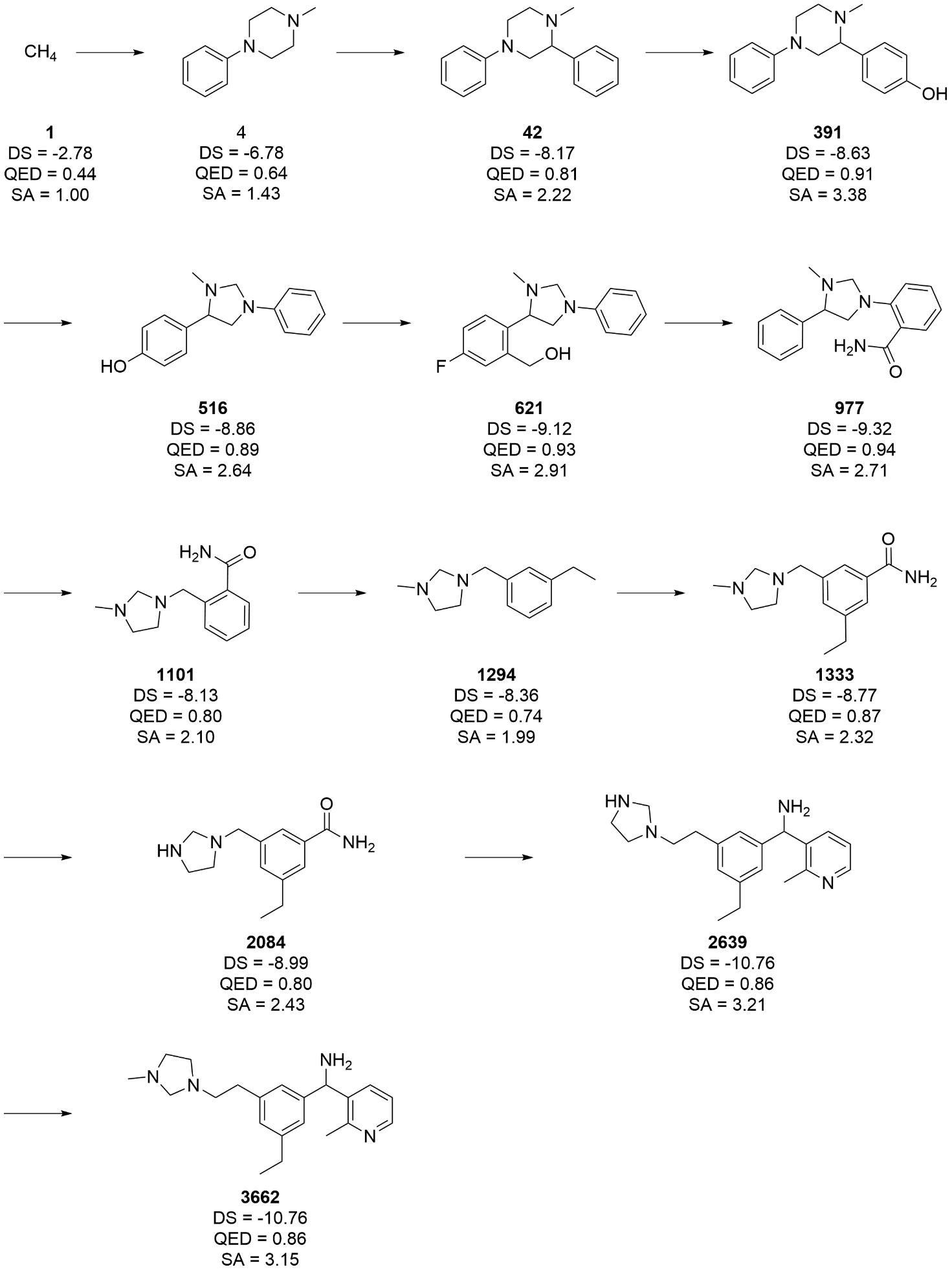


**Figure S9.**

**Figure S10.**

**Figure S11.**

**Figure S12.**

**Figure S13.**

**Figure S14.**

**Figure S15.**

**Figure S16.**

**Figure S17**


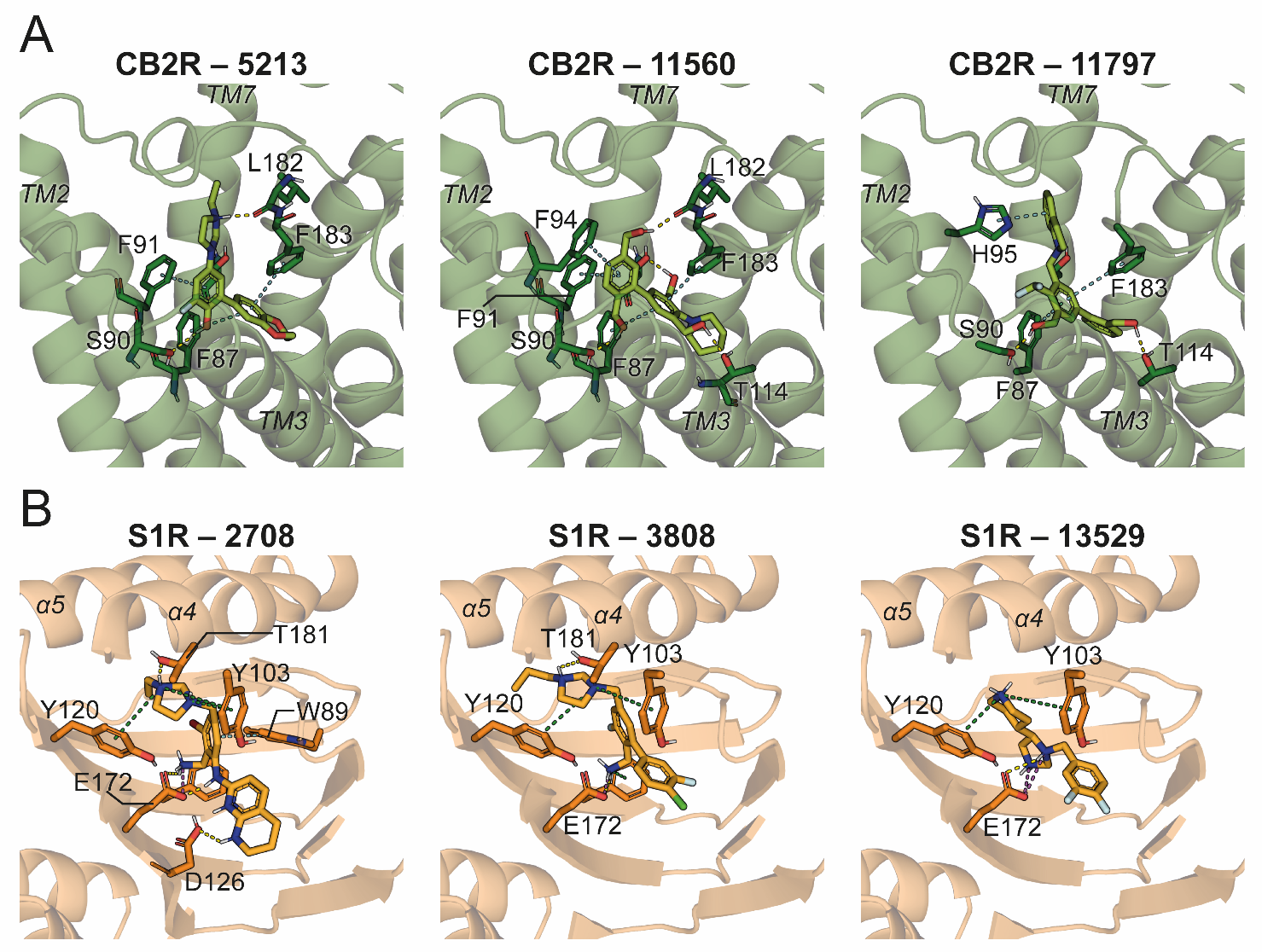


**Figure S18**


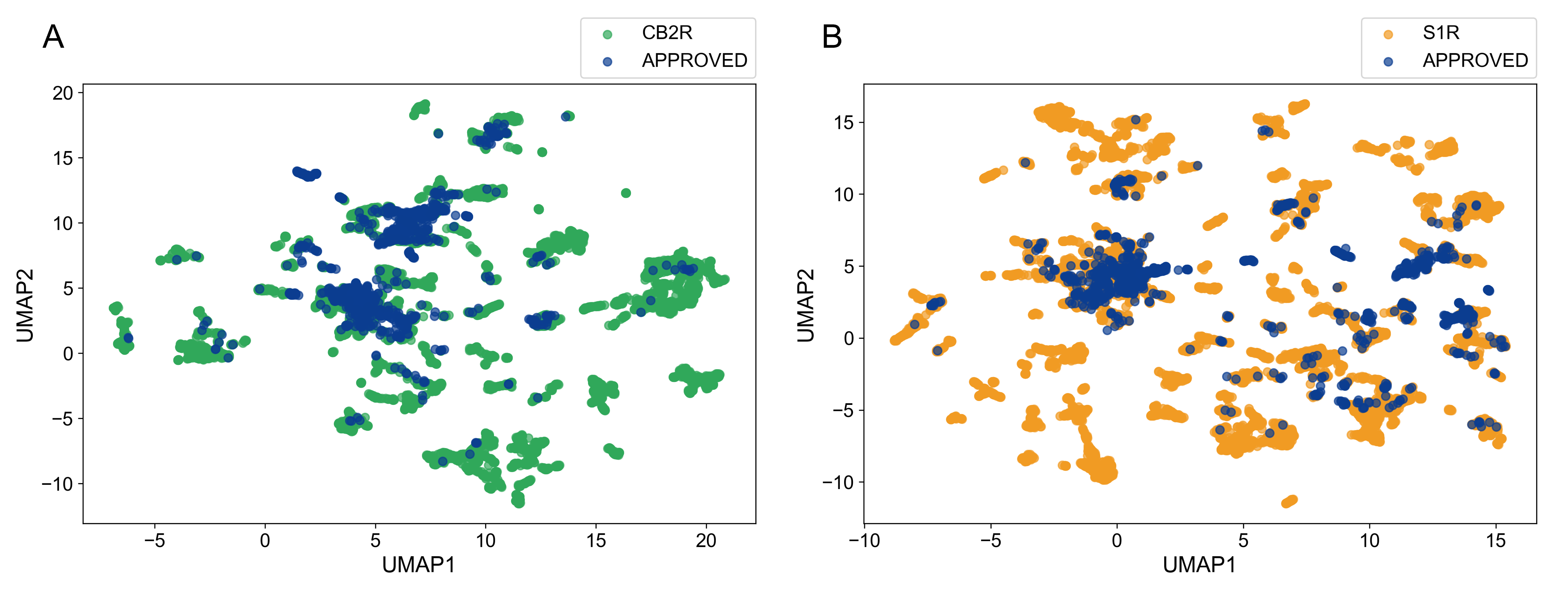


**Figure S19**

**
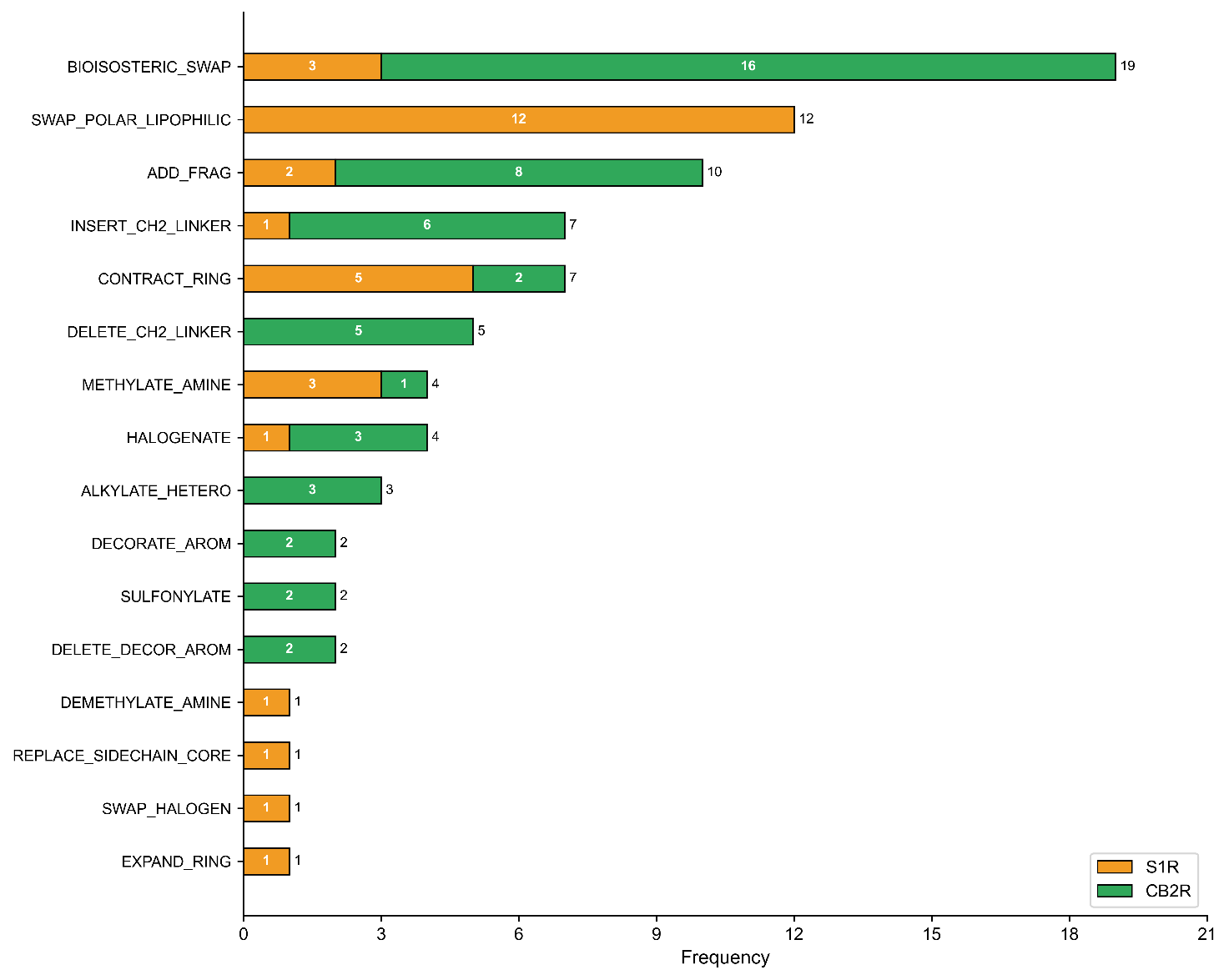
**
